# AAPM WGTEACH Report 366: Best practices in the teaching and mentoring of medical physics

**DOI:** 10.1002/acm2.70259

**Published:** 2025-10-09

**Authors:** Victor J. Montemayor, Elizabeth L. Bossart, Jay W. Burmeister, Ashley Cetnar, Matthew A. Deeley, Jessica M. Fagerstrom, Joanna M. Harper, Kenneth L. Homann, Judy Rose James, Tae Kyu Lee, Harish K. Malhotra, Maria Mamalui, Marija Popovic, Dennis N. Stanley

**Affiliations:** ^1^ Science Department Germantown Academy Fort Washington Pennsylvania USA; ^2^ Radiation Oncology University of Miami Miami Florida USA; ^3^ Karmanos Cancer Center Wayne State University School of Medicine Detroit Michigan USA; ^4^ Department of Radiation Oncology The Ohio State University‐James Cancer Hospital Grove City Ohio USA; ^5^ Medical Physics University of Vermont Medical Center Burlington Vermont USA; ^6^ Department of Radiation Oncology University of Washington Medical Center Seattle Washington USA; ^7^ Radiation Oncology Loughborough University Loughborough UNITED KINGDOM; ^8^ Radiation Oncology Vanderbilt University Medical Center Nashville Tennessee USA; ^9^ Radiology Loyola University Medical Center Maywood Illinois USA; ^10^ Allina Health St. Paul Minnesota USA; ^11^ Dept. of Radiation Medicine Roswell Park Cancer Institute Buffalo New York USA; ^12^ Proton Therapy Institute University of Florida Jacksonville Florida USA; ^13^ Department of Medical Physics The Ottawa Hospital Ottawa Ontario CANADA; ^14^ Radiation Oncology The University of Alabama at Birmingham Birmingham Alabama USA

**Keywords:** didactic, education, mentoring, PER, learning, students, teaching, virtual

## Abstract

Working Group on Teaching Educators and Clinicians How (WGTEACH) has been charged with writing a report to review best, evidence‐based practices in various aspects of the teaching of medical physics. These aspects include not only didactic teaching and mentoring in the classroom and clinic, but also teaching K‐12 and undergraduate students and interacting with the public, including patients. This report also touches on virtual and remote learning, adult learning theory, implications for teaching from neuroscience research on learning, and the role of accrediting and governing agencies in education.

## 1 INTRODUCTION

In 1984, Lillian McDermott published an article in *Physics Today* titled, “Research on conceptual understanding in mechanics.”^1^ In this article, McDermott presented the results of several studies dealing with student misconceptions associated with classical mechanics. The levels of the students in the various studies ranged from junior‐high students through graduate students in physics. The studies discussed attempted to assess student understanding of the concepts associated with the physics equations used in their physics courses.

This 1984 article is seen by many as the beginning of what later became known as *Physics Education Research* (PER). In the article, McDermott made the case that certain types of misconceptions about physics are common to all groups of students, regardless of the level of physics being studied or the ages of the students doing the studying. This article was by no means the first publication in PER, but it was the first paper to pull together the information available at the time and to draw conclusions that were broad and far reaching. Indeed, McDermott acknowledged that a new field of research in physics was emerging, a field inherently different from those of more traditional scientific research studies. She warned of the dangers associated with misinterpretations of results from these studies into student understanding and misconceptions, and the extent to which investigator biases or the degree of interactions between investigator and student can lead to different results. McDermott even provided a summary of characteristics of research on student understanding that would result in more systematic approaches to the research. Such approaches could lead to more valid conclusions about student learning, which might in turn lead to more effective teaching strategies and to the development of more meaningful curricula.

As discussed in the following sections, much work in PER followed McDermott's call‐to‐arms. With the publication of their mechanics diagnostic test in 1985,^2^ later updated to become the Force Concept Inventory,^3^ Halloun and Hestenes provided a quantitative means of assessing a student's conceptual understanding of fundamental ideas in mechanics. In 1991, Priscilla laws used results of the mechanics diagnostic test to show the efficacy of *Workshop Physics*, an approach to teaching calculus‐based physics that was taught solely with student activities and without any lectures.^4^

Shocked at the abysmal results obtained by his students at Harvard on the Force Concept Inventory, Eric Mazur focused his attention on how to improve his students’ conceptual understanding of fundamental physics. The result was the development of the technique called *Peer Instruction*.^5^ Many other physics faculty across the country (and in other countries) were working on alternate ways of teaching physics that leaned away from the passive approach of the traditional physics lecture.

In 1998, Richard Hake published the results of a 6542‐student survey of mechanics diagnostic test/force concept inventory data from physics students in high schools, colleges, and universities.^6^ He also used data from the mechanics baseline test,^7^ which assesses proficiency in more traditional physics problem solving. His analysis of the data clearly showed that teaching methods involving active student engagement during class—as opposed to traditional, passive lectures—can significantly improve not only conceptual understanding of fundamental mechanics, but also the analytical problem solving emphasized in most physics courses.

Just‐in‐time teaching (JiTT) was then developed by Gregor Novak, Andy Gavrin, and Evelyn Patterson.^8^ In JiTT, the instructor assigns reading homework on topics to be covered in class the following day. There is then an online assignment due one‐or‐more hours before the start of class that asks a few open‐ended, conceptual questions on the reading. The instructor then reviews the answers to the questions prior to the start of class and adjusts the time spent on the various topics to emphasize the material with which the students had more difficulty.

Although initially designed for the physics classroom, JiTT is now being used across many disciplines in many countries. In addition, the idea of assigning work to be done at home covering topics yet to be discussed in class ultimately led to the concept of the *flipped classroom* in which the introduction of new material for students takes place at home using typically online materials, leaving more time in class for the active engagement of students with the new material.

It is interesting to note that numerous new approaches to teaching that are used today in many disciplines across the world were originally developed for students in the physics classroom.

The work in physics pedagogy reform resulting from PER did not go unnoticed by the medical physics community. Indeed, members of the community were themselves thinking about how best to teach the various courses in the education of future medical physicists.

The first AAPM‐wide meeting organized to discuss medical physics education was in 1978: “The AAPM Summer School on the Teaching of Medical Physics,” held in Santa Cruz, CA.^9^ Of the 29 sessions held at the meeting, 28 covered topics on *What* to teach. Only one session, held by Perry Sprawls, touched on discussing *How* to teach—more specifically, the approach of teaching facts versus teaching concepts.

The first discussion of alternate pedagogies in the teaching of medical physics took place 30 years later. In 2008, Bill Hendee organized the AAPM Workshop titled, “Becoming a Better Teacher of Medical Physics.”^10^ This workshop was held in conjunction with the 50th anniversary AAPM meeting held in Houston. There were two keynote presentations given at the workshop. The first, “How People Learn Physics,” given by Edward Redish, discussed lessons learned from cognitive science research and how those lessons could be applied to teaching physics.^11^ The second, “Understanding and Engaging Your Audience: Lessons Learned from Undergraduate Physics Education,” by Vic Montemayor, covered alternate pedagogies for teaching physics. This was the first time, results from PER were presented at an AAPM workshop.

In 2009, the *Medical Physicists as Educators Subcommittee* (MPESC) was formed, charged with working to improve the teaching of medical physics. (This subcommittee was changed in 2013 to a standing committee in the Education Council, although it is still known as *MPESC*.)

The 2008 workshop was then followed in 2010 by an AAPM Summer School held at the University of Pennsylvania, again organized by Bill Hendee. The summer school was titled, “Teaching Medical Physics: Innovations in Learning.”^12^ This summer school had a greater emphasis on the use of technology in teaching, but it also had one set of noteworthy presentations from the viewpoint of the use of PER in the teaching of medical physics. Namely, George Starkschall, Jennifer Smilowitz, and George Kagadis each gave a presentation discussing how information on PER presented in the 2008 workshop had been incorporated into their teaching of medical physics. The results of PER were working their way into medical physics classrooms.

Although various symposia and sessions on the teaching of medical physics followed the 2010 summer school, another teaching workshop did not take place until 2018, when Vic Montemayor organized a workshop in conjunction with the 2018 annual AAPM meeting in Nashville. A big change in the education of medical physicists had taken place since the 2010 summer school: in order to become ABR certified, as of 2013 all students had to complete a CAMPEP‐accredited medical physics residency. This meant that many medical physicists were suddenly finding themselves teaching in a medical physics residency program and were not quite sure what to do since they, themselves, had never been in a residency program. As a result, the 2018 workshop, titled “Improving the Teaching and Mentoring of Medical Physics,” emphasized aspects of teaching—or *mentoring*—in a residency program.^13^

As a result of the comments and positive feedback of the 2018 workshop, it was decided to hold teaching workshops every 3 to 4 years, starting in 2023. It was further decided to publish a document summarizing approaches to not only didactic teaching and clinical training in medical physics, but also other aspects of medical physics teaching—for example, teaching undergraduates or educating the public. In July of 2020, the Working Group on Teaching Educators and Clinicians How (WG‐TEACH) was formed with the charge of producing a report on best practices in the teaching and mentoring of medical physics. This report on *Best Practices* is the result of that charge.

It is important to distinguish between what is meant by *teaching* and by *mentoring*. For the purposes of this report, “teaching” will mean imparting knowledge within a structured setting—for example, within a classroom or within the context of a publication or online post. On the other hand, “mentoring” involves the creation of a more personalized relationship between the mentor and the mentee in an attempt to develop the mentee's professional skills and competencies. Teaching is more generalized; mentoring is more personalized. Within the context of this report, mentoring is discussed primarily within the realm of residency training.

This report is structured as follows. Section 2 (Background) forms the report's foundation as it discusses broad ideas in our understanding of how people learn. The section closes with an overview of how medical physicists are educated and trained. Section 3 (Classroom Instruction and Active Learning) addresses the importance of active learning approaches to teaching and provides several means of accomplishing active learning within a classroom setting. Section 4 (Assessing the Effectiveness of Teaching) addresses the difficult topic of assessment, including a discussion of the different classes of assessment along with examples of specific types of assessments. Section 5 (Residency Mentoring) addresses how medical physics residencies can effectively address the three areas of activity of a medical physicist as described by the AAPM. Section 6 (eLearning) discusses the relatively new and growing area of teaching and learning that incorporates various forms of electronic tools and resources for education. Finally, Section 7 (Other Levels of Teaching and Mentoring) addresses the fact that medical physicists do not only teach others within the medical profession—medical physicists must also educate and communicate with K‐12 students, undergraduate students, patients, the general public, and the press.

This introduction has only given a glimpse of teaching practices from an historical point of view. The sections below contain much more detail and greater breadth of coverage of medical physics teaching and mentoring. It should be pointed out that this report does not attempt to prescribe how medical physics should be taught in medical physics classrooms or residencies. Rather, this report presents some approaches to teaching that have been demonstrated to work effectively in a variety of settings and disciplines. It is hoped that you will find inspiration for improving your own teaching in these pages.

## 2 BACKGROUND

### 2.1 Learning theories

As in any field, there are theories that help govern the basic premises of the discipline. Within education there are many learning theories that can be discussed, but we will present three learning theories as an introduction. The behaviorist theory represents learning based on stimulus and response. Using this type of training for education either positively or negatively, reinforces the behavior and assessment is based on the change in response over time. Psychologists Watson and Skinner popularized this theory in the early 20th century within education. Another approach emerged during the 1950s with the rise of cognitive science called the cognitivist theory of learning. In this theory the factors determining learning are not external influences or outside behaviors to be observed but are internal and are based on how the student processes and organizes the information in their mind. Finally, constructivism is a theory in which the student constructs their understanding by adapting new knowledge into their current framework of understanding based on their previous experience. In this view, students are constantly constructing and reconstructing their worlds based on new information where ultimately meaning is derived from their experiences and how they interpret information.

The term “pedagogy” is often used to describe the theory, practice, and methods of teaching. Pedagogy is literally derived from Greek root words meaning to lead children, and indeed, most of our educational practices are derived from classrooms of younger learners. However, it can more broadly describe teacher/subject based instruction. Adult educators such as Malcolm Knowles recognized that adult learners benefited from a more student‐directed instruction. Throughout the second half of the 20th century Knowles developed and refined his theory of andragogy, or the theory of adult learning.^14^ The key tenets of his theory of andragogy include that adults need to know why they need to learn something before engaging in an educational activity. Unlike children, adults have developed self‐concept and are self‐directed. Adults have gained more prior experience throughout their lives developing unique mental models over time based on their experience. In this way this prior experience is a rich resource for learning in the classroom and discussion with others. Readiness to learn is typically need‐based and in response to reacting to a particular task or life situation. In this way their learning is more centered around their life and driven by a desire to develop increased competency and achieve full potential. For adult learners, this motivation is often internal rather than external and education is pursued for intrinsic value to the learner.

Table 1 shows a comparison of the elements of pedagogy with those of andragogy to help illustrate some of the specific differences between the two approaches.^14^ As illustrated by the table, children are much more dependent on the instructor as the source of knowledge, direction, and assessment within the educational environment, while adults typically learn better with more autonomy and have the ability to be more independent in the direction of their learning.

**TABLE 1:** A comparison of the elements of pedagogy with those of andragogy. Adapted from Knowles, Holton, and Swanson (1998).

Element	Pedagogical approach	Andragogical approach
1. Preparing learners	Minimal learner preparation prior to the learning experience	Teachers provide information and help develop realistic expectations. Learners prepare for participation and begin thinking about content prior to learning experience.
2. Climate	Authority‐oriented, formal, and competitive	Relaxed, trusting, mutually respectful, informal, warm, collaborative, supportive, open, and authentic
3. Planning	By teacher	Mechanism for mutual planning of learning experience by learners and teacher in role of a facilitator
4. Diagnosis of needs	By teacher	By mutual assessment by learners and teacher
5. Setting of objectives	By teacher	By mutual negotiation by learners and teacher
6. Designing learning plans	Teacher determines the logic of subject matter and shares information in content units	Teacher presents learning sequenced by readiness and is organized by problem units
7. Learning activities	Transmittal techniques	Experiential techniques (e.g. inquiry)
8. Evaluation	By teacher	Mutual re‐diagnosis of needs and mutual measurement of program by learner and teacher

Teachers involved in medical physics education may educate students at the undergraduate or graduate level and may provide didactic and clinical instruction for trainees in both physics and/or medical residency programs. As such, students may arrive with little or no background experience within the subject matter or a very substantial amount, and educators should prepare their teaching approaches (e.g., pedagogical and/or andragogical) accordingly. In general, a successful leader of either teacher‐directed or mutual teacher/learner‐directed education is one who does more than ask learners to simply remember what they say, or even to do as they say. Learners typically get much more from a teacher who guides them to the learning environment and then allows the learner to navigate their way through it. It should be the responsibility of the educator not only to identify the educational background and needs of the learners, but also to instill critical inquiry and metacognition skills so as to create lifelong learners.

### 2.2 Educational theory and research

A quote attributed to Albert Einstein states that “education is not the learning of facts, but the training of the mind to think.” So, if education is not the learning of facts, why do we devote so much time and effort to this? The late 1800's theory of formal discipline suggested that practicing memorization would increase the faculty of memory, and this would generalize to other activities. Although this sounds plausible (it reminds one of the generalizations of physical exercises to sports activities), we have no evidence that this is true. Much like the practice of medicine prior to clinical trials, much of what we have historically done in education is based on tradition and intuition rather than research, and we are thus susceptible to reasonable sounding but untested practices. Although cognitive science emerged as a field more than 50 years ago, mounting educational research appears to have resulted in relatively modest changes in our educational processes, at least in medical physics. One important aim of this document is to provide educators with the necessary background to create the most effective educational environment, and an understanding of educational research is an important component of this aim.

In the early 20th century, the focus of education was often on acquisition of literacy. However, by the end of the 20th century, the emphasis had shifted to “learning with understanding.” As human knowledge becomes exponentially greater, our ability to cover any reasonable amount of it through traditional education techniques becomes impossible. As stated by Nobel Laureate Herbert Simon,^15^ “The meaning of ‘knowing’ has shifted from being able to remember and repeat information to being able to find and use it.” Despite this important shift in emphasis, one must be careful not to minimize the value of building a factual knowledge base. It is not necessarily the facts themselves, but the meaning these facts carry that is important. Facts in a student's memory that carry no meaning (often referred to as “rote knowledge”) are not transferable and therefore not useful for solving a new problem. However, a substantial amount of knowledge acquired by learners in the process of developing expertise does carry meaning, even though it may be relatively narrow (this has been referred to as “inflexible knowledge”). This is also sometimes referred to as the difference between ‘facts’ and ‘concepts’, the former being information simply memorized without associated meaning and the latter something that is understood. For example, memorization of the ‘fact’ that “E = mc^2^” without any associated meaning is simply rote knowledge. Recognition of the meaning of the quantities in the formula and application to a particular problem makes this inflexible knowledge which has specific meaning to the learner in relation to this problem. Finally, an understanding of the overarching concept of mass‐energy equivalence is flexible knowledge which can be accessed and applied outside of the context in which it may have been learned. Although acquisition of inflexible knowledge may only provide information about the surface structure of a problem, it may be critical in the student's ultimate development of an understanding of the deeper structure of the problem.

An excellent overview of cognitive science is provided within a report from the National Research Council entitled “How People Learn: Brain, Mind, Experience, and School”.^15^ One of the important key findings of this report is that competence requires both factual knowledge and conceptual understanding. The foundation of factual knowledge needs to be understood within the context of a conceptual framework and the student must be able to organize knowledge “in ways that facilitate retrieval and application”.^15^ In other words, what the report terms “usable knowledge” is not the same as facts. A good educator must consider how to facilitate an environment in which students can construct this usable knowledge and apply it to solve new problems. Ideally, such an environment generates, within the student, an awareness of their own thought processes above the subject matter, something often referred to as “metacognition”, or thinking about one's thinking. Metacognition is critical to self‐directed learning, and a valuable tool in the transfer of learning to new tasks or contexts. A key ingredient in developing a metacognitive classroom is providing cognitively active versus cognitively passive activities. This allows students the opportunity to actively transfer factual knowledge into understanding within new contexts. Externalizing the learning process helps both teachers and students understand and optimize the learning process.

There is substantial evidence for the application of cognitive science in teaching reform. For example, research shows that, in the absence of metacognition, a student's ignorance often goes uncorrected because they lack the ability to know whether their answers, or anyone else's, are right or wrong.^16^ The National Research Council report “How People Learn” states that “students come to the classroom with preconceptions about how the world works, and if their initial understanding is not engaged, they may fail to grasp the new concepts and information that are taught, or they may learn them for purposes of a test but revert to their preconceptions outside the classroom.” Active learning techniques such as peer instruction, problem‐based learning, debates, simulations, or case studies have been shown to confer a substantial benefit in overcoming these initial preconceptions. For example, active learning has demonstrated far higher pre‐test to post‐test gains for high school and university students.^17^, ^18^ Cognitive science research not only helps a teacher develop better teaching strategy, but also can guide course logistics to optimize student learning. For example, testing frequency has been shown to have a substantial effect on information recall.^19^ However, while there are numerous studies evaluating the benefits of individual learning techniques (e.g.,^20^), we are not yet translating cognitive science into education at nearly the same rate that biological knowledge is being translated into medical practice. In summary, familiarity with educational research helps teachers better understand the way their learners learn and helps them develop and optimize their teaching strategies. A tremendous amount of scientific information on how people learn is available to us, and it is up to us to identify and implement this information if we are to improve our capabilities as educators.

### 2.3 Medical physics education and training

Education and training for a career in medical physics requires both the development of a strong foundation for scientific inquiry and the preparation in the practical applications of medical physics. In the early days of the application of physics to medicine, most activities were related to research and development of new procedures and technologies. As the development of clinical hardware and software technologies by commercial vendors has become common, the role of the medical physicist has transitioned to include a larger component related to the safe and effective implementation of existing clinical technologies. Curricular recommendations for education and training in medical physics have transitioned along with this change in practice. Although the career emphasis will ultimately be determined by the trainee, the scientific background necessary for research and development work is important even for clinically oriented trainees, and a solid understanding of clinical applications provides context and relevance for the scientific inquiry of the research‐oriented trainee. Medical physics training must therefore provide not only the unique knowledge and skills that make trainees valuable to medicine, but it also must cultivate the critical thinking skills necessary to solve new and previously un‐encountered problems.

The most common educational pathway into a medical physics career is through a graduate degree in medical physics. AAPM Report #365 provides recommendations for the curricular structure of graduate education programs in medical physics.^21^ Those pursuing an academic or research career will most commonly choose to complete a PhD while those pursuing a clinical career may choose either an MS or PhD. There is also an alternative pathway which commonly begins with a PhD in a related scientific discipline followed by the completion of medical physics “core” coursework described initially by AAPM Report #197S^22^ and updated by Report #365. Alternative pathway entrants often complete a graduate certificate in medical physics and recommendations for this pathway and for certificate programs are provided by the Report from AAPM TG‐298.^23^ The standardization of the medical physics education and training pathway allows greater consistency in the breadth and depth of competency of our graduates and trainees. However, the unique knowledge and skills that alternative pathway entrants bring into our profession are critical to sustaining our role as innovators in medicine.

Practical training for medical physicists pursuing clinical careers commonly includes a residency training program which consists of a minimum of two years of clinical training. An accredited residency training program is required for those trainees seeking eligibility to become board certified by the American Board of Radiology. The residency training program must be in the same medical physics specialty in which the trainee is seeking board certification. These specialties include therapeutic medical physics, diagnostic medical physics, and nuclear medical physics. AAPM Report #249 provides recommendations for the curricular structure of clinical residency training programs in each of these areas of specialty.^24^ One additional option is the Professional Doctorate in Medical Physics (DMP). The DMP includes the same core curricular elements as the MS and PhD in Medical Physics, but also includes additional elective coursework and/or research, along with the 2 years of practical clinical training recommended within AAPM Report #249.

Training in research and development skills is an important element in preparing for the future of our profession. Indeed, the AAPM description of the role of the medical physicist states that “medical physicists play a vital and often leading role on the medical research team.” This training includes both basic and clinical research as well as general problem‐solving skills required by the medical physicist. To assure the highest quality clinical research, medical physicists should be substantially integrated into clinical trials research in the core disciplines of practice. In addition, contributions of physicists to medicine are often driven by understanding and training outside of traditional core areas of practice. Instilling critical thinking and lifelong learning skills allows medical physicists to continue to enhance their ability to contribute to the science of medicine.

The AAPM provides^25^ requirements that define a qualified medical physicist (QMP): “For the purpose of providing clinical professional services, a QMP is an individual who is competent to independently provide clinical professional services in one or more of the subfields of medical physics. A QMP is qualified to practice only in the subfield(s) in which they are certified.” The subfields of medical physics include Therapeutic Medical Physics, Diagnostic Medical Physics, Nuclear Medical Physics, Medical Health Physics, and Magnetic Resonance Imaging Physics. A QMP meets each of the following credentials: (1) “Has earned a master's or doctoral degree in physics, medical physics, biophysics, radiological physics, medical health physics, or equivalent disciplines from an accredited college or university”; and (2) “Has been granted certification in the specific subfield(s) of medical physics with its associated medical health physics aspects by an appropriate national certifying body and abides by the certifying body's requirements for continuing education.” The International Organization for Medical Physics (IOMP) similarly defines the clinically qualified medical physicist (CQMP) as a professional medical physicist who is competent to participate actively in the individual clinical field independently.

Specific information on medical physics practice, education, registration, and licensure are available on the AAPM website.^25^ Accrediting organizations assure, through formal peer review processes, that institutions meet minimum standards developed for medical physics education and training programs. Accreditation for both graduate education and residency programs in North America is provided by the commission on accreditation of medical physics education programs (CAMPEP). Certification represents the recognition of the appropriate knowledge and skills of an individual for competent practice in a specific area. A number of certifying bodies are commonly recognized in North America, including the American board of radiology (ABR), the American board of medical physics (ABMP), the Canadian college of physicists in medicine (CCPM), the American board of science in nuclear medicine (ABSNM), and the American board of health physics (ABHP). The certifying bodies that determine the credentials of a QMP are: Therapeutic medical physics‐ABR, ABMP, CCPM; diagnostic medical physics‐ABR, ABMP, CCPM; nuclear medical physics‐ABR, ABMP, CCPM, ABSNM; medical health physics‐ABR, ABMP, ABSNM, ABHP; magnetic resonance imaging Physics‐ABR, ABMP. Finally, registration and licensure are required for clinical practice in some states in the US.

Continuing professional development (CPD) and continuing medical education (CME) are important mechanisms for assuring continued competency in professional practice. This typically involves participation in educational and scientific activities and is a component of some certification programs, such as the maintenance of certification program offered by the ABR. An important aspect of both education training programs and CPD/CME initiatives is to prepare participants and programs to adapt to future changes in the scope and nature of medical physics applications. The goal of these processes is to equip students, trainees, and practicing medical physicists with the education and skills necessary to contribute to, and be leaders of, future advances in medicine.

## 3 Classroom Instruction and Active Learning

### 3.1 Overview

Teaching is modeled to us from a very young age. In the Western world, we are educated within the classroom starting around the age of five, and as professionals in medical physics, we continue to spend time in the classroom for at least the next two decades. Because many of us feel at home within academia, the assumption is oftentimes the best educational approaches have been demonstrated by past teachers, and we are experts at education by osmosis. It is common to teach the way that we have been taught without questioning our own teaching philosophy or methodology.

The traditional approach to teaching via lecture is popular because this was most likely the way we were taught, and lecturing was an effective way for us to learn when we were students. Even though this method of teaching has been in place for generations, there are disadvantages of using a didactic approach that is teacher centric. Using this method, the students are typically viewed as passive receptors of information to be filled by the knowledge of the instructor. A teacher's one‐size‐fits‐all lecture often does not address the differences in individual students within the class. Traditional teaching often encourages memorization, putting less emphasis on student‐focused learning, critical thinking, and process‐oriented learning. Although lecturing can be an effective approach for conveying information in a limited amount of time, it oftentimes bypasses the value of having a classroom environment for active learning and the opportunity to leverage the social capital of the learners. Because this passive method lacks interaction between the instructor and the student, it can be challenging to engage students in ways that will prepare the student for realistic challenges in the future.

In this section, we present an introduction to active learning methods for classroom instruction. Although there is not a single approach that will be perfect for your teaching, the goal is to present several effective evidence‐based teaching methods that you may not have personally encountered in your learning journey as a student. Although there is much in the educational literature about these approaches, we present peer instruction, problem/project/practice‐based teaching, and flipped learning as examples of techniques that can be used.

### 3.2 Peer instruction

One of the challenges in traditional classroom teaching is how lecture material is presented by the instructor. The lecture is often delivered with material directly from textbooks and/or lecture notes, making it difficult to hold students’ attention for an entire lecture. A 10⁠–15 min attention span has been shown to be characteristic of modern students,^26^, ^27^ making it difficult to promote an effective learning environment when lectures are scheduled for an hour or more. This format also forces students to master lecture material outside of class individually, namely through self‐explanation. Peer instruction (PI) was first introduced and popularized by Mazur in 1990's in an attempt to increase the attention span of his physics students by transforming the learning environment through engaging them in active learning during the lecture period.^5^

Crouch et al.^28^ stated that “the goal of PI is to transform the lecture environment so that it actively engages students and focuses their attention on underlying concepts.” The article recommends that each conceptual point in a lecture take roughly 15 min to cover using the following summarized format:

1. Lecture (7–10 min)
2. Short conceptual questions (5–8 min)
  - Pose a question (1 min)
  - Time for students to think about the question (1–2 min)
  - Record and report initial answers (<1 min)
  - Discuss answers with neighboring students (2–4 min)
  - Record and report revised answers (<1 min)
  - Provide feedback to teacher and collect data from students (<1 min)
  - Explanation of the correct answer (2+ min)

Various researchers have found that PI improved student performance as well as comprehension. Giuliodori et al.^29^ tested PI during a physiology course where each 90‐min class was divided into 4–6 short segments within the session. The 15⁠–20 min blocks included a qualitative problem‐solving scenario that could be answered with by multiple‐choice or true/false responses. The results showed that PI improved student scores from 59.3% to 80.3%. Versteeg et al.^30^ performed a randomized comparison of PI with self‐explanation, and found that PI outperformed self‐explanation, 35% versus 23% for the improvement of comprehension of the physiological concept being tested. In addition to increased scores and comprehension, Tullis and Goldstone^31^ reported that students became more accurate and confident when taught using PI.

How do we implement the concept of PI in medical physics classes? Most topics in medical physics lectures require basic knowledge of physics as well as mathematics, biology, and engineering. The commonly recommended forms of PI questions are those based on research and/or teaching knowledge about common ideas that students are likely to have about the topic. Rao and DiCarlo^32^ recommend implementing PI by increasing the complexity level of questions within the same lecture period to help students efficiently follow the progression of the topic and concepts. Not all of the questions require the same amount of time with less time allotted for simpler questions. Examples of questions for each level are presented below on the topic of brachytherapy.

Level 1: Simple recall questions to test the student's ability to recall information.
  Example Q1. (*Conceptually understanding Inverse square law*)
  The intensity of the radiation, i.e. exposure, at 1 m from the source is 100 mR / hr. The exposure ________ at 0.5 m and _________ at 2.0 m.
    - increases, decreases
    - decreases, increases
    - remains unchanged, remains unchanged
Level 2: Questions to test intellectual skills that assess the student's comprehension.
  Example Q2. (*Calculating inverse square law*)
  The intensity of the radiation, i.e. exposure, at 1m from the source is 100 mR/hr. The exposures at 0.5 m are _________ and at 2.0 m __________.
    - 400 mR/hr, 25 mR/hr
    - 25 mR/hr, 400 mR/hr
    - 100 mR/hr, 100 mR/hr
Level 3: Questions to test the student's synthesis and evaluation skills.
  Example Q3. (*Applying inverse square law to known quantities and units*)
  The exposure rate constant of an Ir‐192 source is 4.69 R·cm^2^/mCi‐hr (or 0.469 R·m^2^/Ci‐hr). For 1.28 Ci Ir‐192 source, what is the radiation dose at 10 cm in water from the source with 6.0 min dwell time? Assume that 1.0 cGy = 1.0 R and f_air/water_ = 1.0.
    - 0.1 cGy
    - 1.0 cGy
    - 0.6 cGy
    - 6.0 cGy
  Example Q4. (*Applying inverse square law to known quantities and units*)
  In order to achieve the same radiation dose as that from Q3 at 5.0 cm from the same Ir‐192 source, how long should the dwell time be? Assume that 1.0 cGy = 1.0 R and f_air/water_ = 1.0. (Hint: you don't need to know the answer to Q3 to answer Q4!)
    - 6.0 min
    - 3.0 min
    - 2.0 min
    - 1.5 min

### 3.3 Just‐in‐time teaching

Just‐in‐time teaching (JiTT) is an active learning classroom strategy requiring students to answer one or more open‐ended questions to prepare for teaching activities. Student responses are submitted online in advance of the class, so that the teacher has the opportunity to review and use the responses as a type of formative assessment “just in time.” Teachers then use student feedback to encourage engagement in class, by referencing student quotes and thinking. Consider example JiTT question prompts (all examples quoted from Novak and Patterson, 2010)^33^:

*Introductory physics: Let's say you have a prescription for contact lenses of −2 diopters. If you accidentally get glasses (as opposed to contacts) made to the same strength, your prescription will be a bit off, and you won't be able to focus at infinity. Estimate how far you will be able to focus*.
*Introductory biology: Allison is driving with her parents, Kate and Bob, when they get in a serious car accident. At the emergency room, her doctor (you) tells Allison that her mother is fine, but her father has lost a lot of blood and will need a blood transfusion. Allison volunteers to donate blood, and you tell her that her blood type is AB. Bob is type O. (a) Can Allison donate blood to Bob? Why or why not? (b) Allison, who is a biology student, begins to wonder if she is adopted. What would you tell her and why?*
*Logic: The people of Finiteland only tell the truth on Sunday, Tuesday, and Thursday. Which day of the week is it if a person from Finiteland says, “I told the truth yesterday”?*

JiTT pedagogy was initially developed by Gregor Novak, Evelyn Patterson, Andrew Gavrin, and Wolfgang Christian^34^ in the late 1990s to bolster student learning in college‐level physics classes at the United States Air Force Academy, Davidson College, and Indiana University‐Purdue University Indianapolis. Since its early introduction in physics, the National Science Foundation has supported its implementation for a variety of institutions and study areas. At the heart of JiTT pedagogy are the “warm‐ups” or “pre‐flights” conceptual exercises and question prompts,^35^ and their influence on class time.

In practice, a JiTT‐based lesson cycle will often include the following elements^36^:

1. The teacher develops thoughtful JiTT prompts.
2. Students prepare by completing assigned reading or other assignments.
3. Students read and respond to JiTT question prompts online by a deadline (typically at least few hours prior to synchronous class time).
4. Teachers review student responses for understanding and alternative conceptions and adapt planned in‐class instruction based on feedback.
5. In class, teachers show a subset of deidentified student responses.
6. Following a discussion of student prompt responses, students engage in an activity related to the JiTT content.
7. Finally, teachers plan the subsequent assigned work and JiTT prompts based on material covered during class time.

When implemented as intended, JiTT should not be just another form of traditional homework assignments or take‐home exams. The goal of the JiTT process is not to assess what students have already learned (as is commonly done with traditional take‐home assignments), but instead, to prompt students to activate prior knowledge, to think deeply and critically about open‐ended questions, and to use student responses to inform and adapt in‐class activities. It is key that educators not assign interactive exercises as busy work, which will eventually foster resentment and discourage student engagement.^37^ JiTT may be used in conjunction with other active‐learning strategies,^38^ including peer instruction and flipped classroom. Some of the last‐minute preparation required for successful JiTT implementation will be new to educators who are used to more traditional methods, allowing instructors to adapt and respond to student thinking on‐the‐fly.

Though JiTT was initially developed for undergraduate introductory physics courses, JiTT may also be used in a variety of classroom and educational environments with access to online submissions through a learning management system. Published literature has detailed its implementation for a wide range of learning contexts: see, for example, JiTT in the following courses: high school math and science,^39^ undergraduate mathematics^40^ and physics,^41^, ^42^ medical school,^43^ university‐level science education,^44^ graduate‐level architecture,^45^ and within professional radiography curriculum.^46^

Education research has indicated the effectiveness of JiTT in the classroom. A comparison of first‐ and last‐day answers of normalized gains of multiple‐choice questions may be used to assess student learning, as described by Hake when studying over 6000 introductory undergraduate physics students.^47^ Using this metric, compared to traditional teaching methods, classrooms employing JiTT pedagogy showed significant improvement in cognitive gains in undergraduate biology,^48^ chemistry,^49^ and physics^50^ courses. JiTT has also been shown to decrease failure rates,^51^ decrease attrition rates,^35^ and improve student perceptions of learning.^42^, ^52^

It is noted that direct instruction or interactive direct instruction,^53^ sometimes known as just‐in‐time *learning* or just‐in‐time *instruction*, denotes a different practice from JiTT. Interactive direct instruction describes lecturing at strategic points to share ideas at the conceptual level too complex for students to work out on their own. This strategy is often used to introduce a laboratory exercise, with the goal to support students in sense‐making. In general, interactive direct instruction is not as productive for student learning if it is used to explain a natural phenomenon such that students can reproduce and confirm that phenomenon in the lab through a set of predetermined steps.

For medical physicists involved with classroom learning, JiTT can represent a helpful tool to encourage active learning by students. Well‐designed JiTT question prompts will of course vary widely based on student level as well as class learning objectives, but some possible examples include:

In your own words, describe the Central Slice Theorem.
In modern intracavitary gynecological brachytherapy, do you think reporting Point A and Point B doses is useful? Why or why not?
A linear accelerator's pressurized monitor chamber develops a leak during annual QA. The temperature in the vault gradually warms up during scanning measurements. How does the output of the linear accelerator change as a result?
In radiation therapy shielding design, when calculating the barrier transmission factor for primary barriers, what is “workload”? What factors are important when determining the workload?
If you could design an ideal radiotracer for use in nuclear medicine, what would its characteristics be? Why?
A precocious six‐year old asked you how ultrasound images are generated. How would you answer this question?

### 3.4 Problem, project, and practice‐based learning

“Tell Me and I Forget; Teach Me and I May Remember; Involve Me and I Learn.”

‐Chinese Proverb

In the modern world with many distractions, it often becomes difficult to maintain the learner's interest during the entire duration of a class, challenging the value of traditional teaching approaches. Project, problem, and practicebased learning methods employ active learning strategies showing better outcomes in meeting learning objectives, improving long‐term retention, and providing opportunities for development of practical skills indispensable in career development. Although they all share the same acronym (PBL) and are student‐centered, there are differences in the ways the active learning strategies have been incorporated with each method.

#### 3.4.1 Project based learning

Project based learning (PBL) organizes student learning around projects. This is an active learning student‐centric approach where students learn by working on complex projects. The students usually work in small groups to solve a problem with little or no initial guidance or suggestions from the teacher. This requires students to work together to develop a plan and they could end up spending far more time and energy on their work than originally intended by the instructor. Students, therefore, can retain knowledge and develop problem solving skills more effectively than if they had been simply told how to complete the project by their teacher. PBL allows students an opportunity to work relatively autonomously over extended periods of time with the traditional role of the classroom teacher essentially becoming more like that of a research mentor. The fulfillment involved in successfully completing a project not only provides much better learner satisfaction but can also affect changes in their future professional practice. Working collaboratively on a project also helps in developing interpersonal skills and teamwork, which will be helpful in their professional career.

Examples of medical physics projects that can be used in PBL are selecting QA equipment in a new clinical setup, application of 3D printing in the clinic, or the room design of a linac with shielding computations [https://www.aapm.org/education/vl/vl.asp?id=12661]. Many medical physics projects are possible in a clinic, e.g., the application of adaptive radiotherapy in a clinic, suitability of MR‐linac in radiotherapy, motion management in radiotherapy etc. are some of the examples of such projects which can be easily implemented in project‐based learning.

#### 3.4.2 Problem based learning

Problem based Learning is a student‐centered pedagogy in which students learn about a subject by the experience of solving real‐world problems and in the process gain knowledge. Historically, problem based learning started at Case Western University in the 1950s with the goal of providing real‐world problems for medical students to solve. Other medical schools and eventually business, law, science, engineering, and education fields quickly embraced this method of teaching as well.

One of the strongest attributes of problem based learning is that it can be implemented in almost any field and the students have independence and autonomy during the educational process. In problem based learning, the students are presented with an ill‐structured problem, where “ill‐structured” is defined as meaningful to students in the real world but may not have a straightforward answer or may even remain open‐ended. The students, therefore, must think critically above and beyond the curriculum to apply knowledge in new situations. The thought process, strategies adopted, and research carried out behind a solution are more highly valued than the actual final solution in this methodology. Problem based learning may not yield a final product like project based learning. Possible examples of problem based learning could be, “What would be an optimal treatment energy for a clinical linear accelerator?”, or “If your team could manufacturer only one radionuclide, which would you select for nuclear imaging applications?”

As per Paulina Nasloski,^54^ problem based learning can be implemented at the curriculum level, group level, or individual student level. At the curriculum level, all problems and objectives are identical for all students, and yet each student may develop their own individual solutions to the ill‐structured problem presented. At the group level, each group can be presented with combinations of different problems and objectives for the topic. Lastly, at the individual student level, the instructor can predetermine objectives for each individual student.

#### 3.4.3 Practice based learning

As the name implies, practice based learning disseminates knowledge by combining high‐quality course content with opportunities to practice, which is now the standard method of teaching in healthcare. Research has shown that lectures alone have little significant or long‐term impact, either on a learner's professional practices or on improved patient outcomes.^55^ A medical student/resident is involved in education by didactic lectures, obtaining patient clinical history, ordering pertinent medical tests/medicines, shadowing the clinical decision‐making process with his/her attending, and learning through meetings such as chart‐rounds and morbidity and mortality (M&M) conferences. They may even be operating on a real patient under the watchful eyes of an attending.

The student first learns the skills through study, watches it in use, hones the skills by practicing on real patients, and refines it based on feedback and evaluation from their mentor/attending. Based on performance and competence, more and more opportunities and responsibilities are provided to the student so that by the final year of medical college/residency, they are consistently meeting the expectations of a new attending in their professional career. In practice based learning, the students do not actually produce a physical product but work on maintaining or improving practice and better identifying and preventing poor practices. Sensing its importance, the Accreditation Council for Graduate Medical Education (ACGME) is now advocating for the importance of incorporating practice based learning into medical residency curriculum by having a formal competency evaluation.

Practice‐based learning is also integral to medical physics education, offering students and residents opportunities to develop essential skills, demonstrate competency, and engage in reflective practices to ensure clinical success. Although medical physics residency programs inherently incorporate practice‐based learning, similar methodologies can be adapted for graduate‐level training. For example, a student's competency in performing quality assurance tests and evaluating patient chart reviews can be assessed under the guidance of a qualified medical physicist. Furthermore, exposure to complex case studies can be used to foster the application of problem‐solving strategies for addressing technical challenges that may not typically arise in standard clinical rotations. In addition, given the importance of communication in clinical practice, structured exercises—such as interactions with standardized patients—can be used to serve as effective approaches for refining communication skills among future medical physicists.

#### 3.4.4 Conclusion

Even though all of the PBL methods introduced are primarily planned and driven by students, a teacher still plays an important role by determining specific learning goals, establishing academic rigor at various stages, and establishing timelines and rubrics for the evaluation. The teacher who has been the star of the class in a traditional lecture‐based education system essentially takes over the responsibility of a mentor/advisor/coach in the new learning paradigm. The time commitment of the teacher is less devoted to creating lecture material and grading and can be focused on developing quality problems/projects/practices and providing feedback to students.

Challenges to this technique include scaling methods for large classes and can complicate learning evaluation, as the students do not produce traditional assessment results. In collaborative work, it can be difficult to evaluate each student's contribution objectively and fairly as team members distribute the project/problem and perform different tasks where some might be easier/less mentally/physically taxing than others. The challenge of carefully developing the PBL experiences is important—the students should be led to learn and grow but remain motivated to complete the PBL. Despite the effort required, the methods presented in this section can better prepare a student for their professional career by providing authentic educational experiences.

### 3.5 Flipped learning

Robert Talbert in his 2017 book Flipped Learning: a Guide for Higher Education Faculty^56^ said, “Flipped learning is a pedagogical approach in which first contact with new concepts moves from the group learning space to the individual learning space in the form of structured activity… and the resulting group space is transformed into a dynamic, interactive learning environment where the educator guides students as they apply concepts and engage creatively in subject matter.”

So, what are the elements of a flipped classroom? In a so‐called “flipped” classroom, students are first exposed to learning a concept prior to class using carefully curated sources of knowledge.^57^ For example, in a class about radiation protection when focusing on regulatory issues and what it means to be an agreement state, sourcing videos by the Nuclear Regulatory Commission on radioactive source materials security (https://youtu.be/bOa5KFKaiAE), another on NRC partnering with state regulators for agreement state programs (https://youtu.be/o58YO‐O2I5I), and perhaps pointers to the relevant state and national regulations as background material can be provided on the topic for students to view prior to coming to class.

Having the students go through the basic material prior to class allows the students to focus on the processing part of learning with the support of the teacher and their fellow students. In‐class time can focus on a deeper dive into the material rather than presenting the surface facts. Going back to the radiation protection class example, in‐class time could be spent on a discussion of what it means to keep sources secure in various types of clinics (nuclear medicine vs. radiation therapy) or a policy discussion of positive and negative aspects of being an agreement state. After class, the students complete activities to reinforce the in‐classroom experience.^57^ In our radiation protection example, that could be writing a policy position paper as a consultant for a state that is not yet an agreement state on reasons why or why not to become an agreement state.

This series of steps is opposite the traditional classroom where a student's first exposure to new concepts is via a classroom lecture, and students are expected to synthesize their learning via assignments outside of class. Though the up‐front labor to make a flipped classroom function properly can seem daunting, it can be a satisfying experience for both teacher and students if done well. Such changes allow the instructor to better monitor student learning and encourage the application of the content in ways that help students to develop their skills as problem solvers during scheduled class time.

Simonson in the discussion article “To Flip or Not to Flip: What are the questions” proposes there are several factors that must be considered when deciding whether or not to flip a class either in part or completely.^58^ First, the complexity of the course content as well as the expertise of the students taking the class should be taken into account. Then ask the question of how “flipping” will benefit your students.^59^ If the goal is just to expose students to material, meaning they only need to remember and understand the content, then flipping a classroom may not be ideal. If they to need to analyze, evaluate, or create from the information provided as result of the class, then flipping the classroom may allow students to build the scaffolding of knowledge outside of class and raise the building inside the classroom with full support of teachers and peers.

The second idea to consider is situational factors out of the control of the teacher.^58^ Institutional norms may make it difficult to try new educational methods in the classroom. The format or size of the classroom may make flipping a class difficult. The time in which an educator has to prepare for a new class format can also play a role in whether or not preparation for flipping can be organized.

The next consideration is the students themselves. Students who have never worked in a flipped class format may be resistant to the technique. It is important to set expectations for the class at the beginning of the semester so that this is the norm instead of changing methods after a teaching pattern has been established mid‐semester. The up‐front work they need to do before class may be unfamiliar. That work by the students, however, generally comes with the pay‐off of less test‐prep for exams.^60^, ^61^, ^62^, ^63^ As they have worked hard to gain the knowledge, but in a supportive environment, students generally feel more comfortable with complicated materials and less stressed by exams through the semester. However, convincing students used to passive models of learning this active learning model is worth the effort may not be easy. With a little time, the classroom interactions will hopefully overcome initial hesitancy with the flipped classroom environment. Students may need some form of incentive to prepare, but such activities should be meaningful in a way that furthers the educational process.

The final consideration is probably the most important – instructor factor.^58^ Flipping a classroom necessarily hands over some of the control of the classroom to the students. The use of this new format should be meaningful to the instructor as well. If the instructor is unable to see how this change will fit into their goals, then making such a change will feel uncomfortable for both the instructor and the students. Designing feedback into the curriculum for assessment will help the instructor to monitor how the transition is going and provide encouragement. It is also recommended to investigate what resources are available in your local institution for instructional support including new teaching ideas and lessons. The librarians in your institution may have access to resources or the ability to find new resources that can be added to the pre‐preparation for the classroom.

The next step is creating active engagement in the classroom to build on the pre‐class planning and work done by the students. Guillaume, et al. have written an overview of many different ideas for active engagement in the classroom that can be used in the flipped class format,^64^ and Jennifer Baumgartner, an Associate Professor at Louisiana State University has an active Google document with teaching tools that can be used for mixed synchronous and/or asynchronous learning environments where synchronous sessions take place with all learners coming together (either in person or virtually) at the same time and asynchronous learning occurs in the same period of time for the learners (e.g. over a week) but not necessarily simultaneously.^65^ Playing games to quickly test the knowledge of the students, hosting debates, or getting students to work together on problems can help with engagement. Provide time for questions and discussion of deeper ideas around the material within the classroom.

Using pre‐recorded lectures as educational materials to use as asynchronous resources for supporting the flipped classroom can provide additional time for active learning activities during synchronous class time. If you are looking to record your lectures for this purpose, Katie Ash suggested the following in her article^66^:

1. Do not worry so much about creating your own videos.
2. Be thoughtful in what parts of your class you decide to flip as well as when.
3. If possible, find a partner with whom to create videos.
4. Make sure to address any issues of access early so that you can be sure that all students are able to reach the materials to be used outside of class.
5. Find interesting ways to engage students in the video.

Keeping video materials short and focused helps engage students and allows them to fit materials into overworked schedules. It is generally simpler, both for you and them, to have 3–4 self‐contained, 15‐min video lectures rather than to have them sit through a single hour video.

If you are interested in flipping a class, keep in mind when trying out this technique that it is not necessary to flip a class all at once. Instead, try picking a lesson or two in which students typically struggle. Give students a short video, pre‐recorded lecture, or paper to introduce the concept, then use class time to answer questions, discuss concepts in small group discussions, or mentor small groups on the topic.^59^

## 4 Assessing the Effectiveness of Teaching

In order to assess the effectiveness of teaching, it is important to provide opportunities to understand student learning. Although preparation of the teacher is vital to the success of classroom infrastructure, the ultimate goal of teaching is the success of the learning outcome. There are many different assessment techniques, which can be used by a teacher to better understand the effectiveness of their instruction.

“In clinical medicine we use certain principles to diagnose a disease. First, we don't rely on a single ‘test’ result. Second, we don't combine information from different results within the same instrument, for example compensating for a high glucose level with a low sodium level, just because they are both laboratory values. And, finally, we don't make high‐stakes decisions based on only one test.”^67^ This quote compares the evaluation of patient health with the evaluation of student learning. In the clinic, not just one set of test results is used to make important decisions for patient treatment. Instead, we use multiple instruments and their associated results to make on‐going clinical decisions. As this is an important idea in medicine, it is also applicable to the classroom in the assessment of student learning.

### 4.1 Definitions

Swanwick, Forrest, and O'Brien define assessment in medical education as “any *purported* and *formal* action to obtain information about the competence and performance of a candidate.”^67^ Although not all assessments need to be “formal action,” the specificity and inclusion of competence and performance are additional aspects of assessment that should be considered. Competence and performance are tangible aspects that can be observed as potential surrogates of student learning in medicine.

Whether intended or not, assessments shape the culture of the classroom. “The saying ‘students don't do what you expect, students do what you inspect’ epitomizes the educational impact of assessment.”^67^ Having a well‐defined goal and purpose of the assessment are essential. The evaluation should be specific and be used to draw conclusions about student learning. Cunningham is of the opinion that “assessments are always used to make decisions. If no decisions are to be made, no assessment should be conducted.”^68^

A range of assessments is useful in teaching so the instructor can better understand the learners in a more comprehensive way. By using a variety of instruments, the educator can learn about the students’ background knowledge, how they are progressing through their learning during the class, and finally what they have learned because of the program or instruction. Common formats for assessment include diagnostic, formative, and summative assessments to meet these goals.

### 4.2 Diagnostic assessment

#### 4.2.1 Details of Usages

The first class of assessment that is useful for evaluating student learning is diagnostic assessment. This is a way to find out what students already know before commencing with the teaching or learning activity. Diagnostic assessment is one way of attaining a baseline of student understanding before your teaching intervention. Since students are not blank slates, bringing their own experiences and knowledge into the classroom, diagnostic assessments can help you find out what they know in relationship to the domain knowledge that you intend to teach. This way you can find out if your intervention was helpful, or if the students already had experience in your topic.

Diagnostic assessment can “help you identify your students’ current knowledge of a subject, their skill sets and capabilities and to clarify misconceptions before teaching takes place.”^69^ Students already have many ideas about how the world works. However, their understanding may be somewhat inaccurate or incomplete. These misconceptions can be evaluated ahead of time so that these individual barriers can be addressed through the lessons. It has been shown that many students have similar misconceptions in physics, but it is helpful to identify the potential misconceptions of your students.^70^ Statistically validated concept inventories are one type of assessment that are commonly used in introductory physics classrooms for diagnostic assessment.^71^

Because some students may have more experience in particular topics than others, diagnostic assessments can be “used for remediation or academic acceleration.”^68^ A well‐designed assessment can help to judge what level of teaching would be appropriate for individual learners. It can also help place students in an appropriate teaching environment if there are different tracks or course levels offered.

Examples of diagnostic assessments include:

Pre‐tests
Self‐assessments
Discussion board responses
Interviews
Concept inventories

#### 4.2.2 Advantages and drawbacks

Advantages to including diagnostic assessments in your curriculum are that they are helpful in guiding the design of the course or lesson. This initial feedback can steer the instruction in a beneficial direction from the beginning. By having this as a preliminary assessment, you can spend instructional time on the areas of need if they have already demonstrated mastery of a topic. Drawbacks to diagnostic assessments are that it takes time to distribute the assessment. If you use the technique, it then takes time to customize the lessons and the course for each group of students instead of using ready‐made materials or previous notes for the class. If you have information that students are all at different levels, providing differentiation for each level of students in the teaching can also be a challenge. Even though there are challenges as a result of the additional information, it is ultimately to increase the quality of the instruction of the course if the data gathered is applied to teaching and learning.

### 4.3 Formative assessment

#### 4.3.1 Details of usages

Unlike diagnostic assessment, in which the evaluation of student understanding comes before the teaching, formative assessment is a way to help assess your progress as an instructor. The goal of formative assessment is to provide “feedback and information during the instructional process, while learning is taking place, and while learning is occurring.”^69^

Conducting formative assessments can be of great value to the instructor. Results of these evaluations “may include decisions about the rate that content is presented or whether there is a need to repeat what has already been taught.”^68^ Because these checkpoints are designed to be informational, formative assessments do not have to be graded and can be informal ways of collecting data from the class. The goal of these assessments is to provide feedback to you as a teacher to make changes to your plan of instruction throughout the teaching to meet the students where they are.

There are many different methods of formative assessment. Classroom assessment techniques (CATs) have been published and take many different forms. For example, the use of audience response devices or “clickers” to survey the learners can be used to check knowledge or receive feedback throughout the session. With the advancement of technology, there continue to be new ways of teachers eliciting feedback from students through educational apps through smartphones and tablets. As another example, quick writings such as minute papers (writing a short summary of what was learned in 60 s) or muddiest points (what concepts or ideas are still unclear or confusing) can be used to provide feedback to the instructor based on what students found important from the lesson and reveal gaps that still remain. Students can also construct concept maps as a way of graphically organizing understanding using nodes for ideas and lines and arrows with labels associated with relationships between the concepts to help demonstrate the organization of their knowledge.

Examples of formative assessment include:

Observations
Reflection journals
Question and answer sessions
Conferences
In‐class activities
Self‐evaluation
Clickers/Audience response systems

#### 4.3.2 Advantages and drawbacks

Advantages to formative assessments are that they are quick ways to see how individuals are progressing because of the instruction. This is a way for instructors to reflect and redirect teaching to help students learn as a dialogue with the students throughout the class. The benefit is that students are able to reflect and practice in low‐stakes environments and the teacher gains additional information from the results of the assessment to modify teaching. Disadvantages are that it takes time for students to complete these reflections and formative assessments, especially if they are conducted during class time. The instructor also must make time to use the feedback to adjust lessons that take time to prepare before the next session.

### 4.4 Summative assessment

#### 4.4.1 Details of usages

The final category of classroom assessment is summative assessment. This is the most familiar assessment method because summative assessments are so often used for educational evaluation. Summative assessment “takes place after the learning has been completed and provides information and feedback that sums up the teaching and learning process.”^69^ These assessments can be at the end of a unit or course to show a result of the teaching and learning.

Written examinations such as multiple‐choice tests are one way of conducting a summative assessment. However, open response and oral exams can be other methods for providing summative feedback. Rubrics with specific criteria of expectations can be helpful for both the teacher and the learner providing a tool for consistency in grading among students. Summative assessments can be high stakes where the completion of a course, grade, funding, or professional standing could be at stake for the candidate, educator, and program.

Examples include:

Assigning grades
Examinations
Term papers
Projects
Portfolios
Performances
Student evaluations
Determination of whether a student is to be promoted or detained
Whether a student is accepted or rejected by an educational program

#### 4.4.2 Advantages and drawbacks

An advantage to summative assessment is that it can provide overall feedback for the student as result of the learning experience. This is the end product to see if they achieved the learning objectives that were intended. Well‐designed summative assessments can also provide differentiation between student abilities and understanding at that point of the assessment. Unfortunately, these forms of assessments usually occur at the end of a learning experience, and there is not an opportunity or incentive to learn from misconceptions. This can reduce the students’ motivation to master concepts that they may not understand. Summative assessments can be a source of anxiety for students when the stakes are high. Finally, poorly designed summative assessments may not be congruent with what was taught so validity of the assessments should be evaluated before using them in high stakes situations.

### 4.5 Conclusion on assessments

**FIGURE 1:**
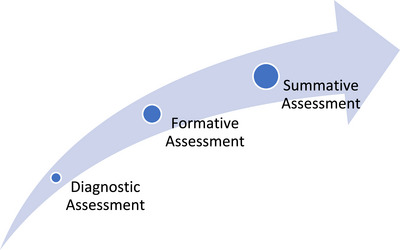
Potential trajectory of assessments in the classroom.

In conclusion, diagnostic assessments can be used by instructors at the beginning of the course to assess where the learners are starting. Formative assessments are a way of receiving feedback about how the learning is progressing as a result of the teaching, and summative assessments are a way of final evaluation to see if students understand and meet the learning objectives. An example of the progression of assessments is shown in Figure 1. This arch can be used over a course or for each individual unit of lessons. Although this figure only shows one time point for formative assessment, this can be repeated several times using different classroom assessment techniques for the learners.

### 4.6 Assessment instruments

Although diagnostic, formative, and summative assessments are different philosophically in why they are administered and what data is gathered in terms of student learning, many different assessment instruments can be used to understand student learning. Although summative assessment has traditionally been the most common use for assessment, evaluations can also be set in low‐stakes environments with the goal of providing diagnostic or formative assessment as well. The three formats presented in this report will be written assessments, objective structured clinical examinations, and oral examinations because they each have strengths for use in medical education.

#### 4.6.1 Written assessment instruments

When students are found in the library feverously reviewing notes and flashcards late at night, there is a good chance that they are preparing for a written assessment. There is an understood format for written exams that students must know the information and be able to answer the questions correctly during a set amount of time. The stakes are usually high for a medical student where the results of the exam could determine whether the individual passes a course, how he or she is situated within their class rank among their peers and may influence whether the student will be able to continue to pursue a particular medical specialty. Types of written assessments include multiple‐choice questions, open‐ended questions, and case‐based questions with each having advantages and disadvantages to their format.

#### 4.6.2 Multiple‐choice questions

Multiple‐choice questions are the staple questions that come to mind when thinking of medical examinations. A question or problem is presented with multiple options, typically labeled *a* through *e* as potential answers for the question. Advantages of multiple‐choice assessment are that they are cost‐effective in the sense that they require very few resources to administer the examination. They are also very easy to score with the use of technology. However, multiple‐choice examinations take more time for the instructor to write quality items, and there is a chance of students guessing the correct answer when options are provided.

When compared to other formats, multiple‐choice tests allow for more questions to be administered in a fixed amount of time, which could allow for the reliability of the assessment to be increased with a greater number of items in the sample. For example, asking 50 questions can provide greater reliability in representing understanding than five questions. Various metrics can be used to assess the items on multiple‐choice tests, including the commonly used *p*‐value, which represents the percentage of students that answer the question correctly.^72^ The A‐value represents the attractiveness of a distractor option which can be useful in refining test questions which can incorporate the frequency in which a specific distractor is selected and can incorporate a second factor by asking the respondent's confidence in their response.^73^ Other metrics include item‐total correlation which describes those questions that strong candidates correctly answer, and item‐rest correlation which is an indicator of the questions weak candidates are answering incorrectly.

#### 4.6.3 Open‐ended questions

Open‐ended questions are a form of written assessment where the students provide the answers to the questions. Unlike the multiple‐choice questions with options provided and a probability of guessing the correct answer, open‐ended questions require the student to generate the response to answer the question. An advantage to open‐ended questions is that they have the ability to test various cognitive skills, but this is dependent on how the questions are asked. One of the greatest disadvantages to open‐ended questions is that they can require a much higher workload to score responses.

When comparing the results of multiple‐choice tests to structured response from the advanced placement (AP) testing, a study by Lukhele, Thissen, and Wainer concluded:

Overall, the multiple‐choice items provide more than twice the information than the constructed response items do. Examining the entire test (and freely applying the Spearman–Brown prophesy formula), we found that a 75‐min multiple choice test is as reliable as a 185‐min test built of constructed response questions. Both kinds of items are measuring essentially the same construct, and the constructed response items cost about 300 times more to score. It would appear, based on this limited sample of questions, that there is no good measurement reason for including constructed response items.^74^

#### 4.6.4 Case‐based

A variation of open‐ended questioning includes case‐based questions. Instead of presenting abstract questions in isolation, this format provides context for the situation in realistic scenarios that are helpful in many professional fields including medicine. Case‐based questions “contain a case description and questions that ask for essential decisions or an evaluation of the problem.”^67^ These questions are generally well received by medical students because of the applicability of the questions to clinical practice. Advantages of case‐based problems are that the questions can evaluate high order thinking such as the “application of knowledge and problem solving.”^67^

Examples can use clinical situations in which problem solving is necessary, such as challenges with treatment planning, machine calibration, or radiation safety incidents. In addition to technically related cases, articles addressing leadership and ethics have recently been published in the Journal of Applied Clinical Medical Physics. A guideline for developing strong cases in medical physics can help instructors implement this teaching.^75^

#### 4.6.5 Objective structured clinical examinations and simulated patients

Objective structured clinical examinations (OSCE) and simulated patients (SP) are popular for the assessment of practical skills in medical education. These assessments are controlled situations that emulate clinical situations under examination conditions.^67^ Standardized patients are carefully trained to replicate a patient encounter and use their bodies as the content to be assessed.^76^ The environment for these assessments is usually configured such that each room or station has a specific task that the student must perform for either a simulated patient or manikin. The progress of students through the stations by a set amount of time or upon completion of the activity can be monitored. Assessment is based on either a checklist of competencies or by a rating scale by a physician evaluator or the simulated patient.

An advantage to the OSCE with SP is that the structure helps provide realistic scenarios that apply student learning with the goal of assessing both clinical and communication skills. This format also replicates the unpredictability of the clinic by mixing different skills and application of knowledge sequentially throughout the assessment. This methodology is currently underutilized in medical physics education as a tool for assessing competency in task‐based procedures, problem‐solving scenarios, and communication with team members or patients. The observations and tasks are short so that you can sample a variety of items, thereby increasing the reliability of the assessment. Although brief, there should be adequate amount of time allowed at each station to demonstrate the skill based on what would be required clinically since not all tasks are equal. Because of the number of stations, you can have many different examiners to help sample across different individual judges. Because these tasks require clinical skill and judgment, it is important to establish the criteria for passing before administering the examination. The OSCE with SP format allows for a degree of standardization of clinical assessment instead of relying on the variability of patients and cases within the clinic.

A disadvantage of OSCE with SP assessments is that checklists can be memorized and passed on from year to year. However, there is less value in memorizing rating scales that can be used as metrics. Conducting OSCE with SP are very expensive, requiring professional evaluators, clinical resources, and logistics for organizing the event. Although providing a grade for these examinations appears to be quantitative, the grading is reduced to either the completion of specific behaviors or the interpretation by the examiner using a rating scale. There can also be variation between simulated patients, including how they are trained and how they act during the examination. Some OSCE with SP are assessed by the standardized patients that are trained as a subject but are not medical professionals. The simulated patients’ perspectives are valuable when assessing skills such as communication. However, the validity of the assessment can be strengthened by using clinicians who are content experts as a third‐party examiner observing the stations.

#### 4.6.6 Oral examinations

Oral examinations have been used since medieval times for educational candidates to demonstrate their level of competence within the university. Oral defenses are still used in the university setting to determine the academic readiness of doctoral candidates for graduation. This method of examination has the advantages of allowing the candidate to teach, explain, and defend their understanding.

In medicine, oral exams are helpful for assessing problem‐solving and reasoning, probing depth of knowledge, assessing flexibility in moving from one topic of expertise to another, being able to tailor questions asked to the needs of the individuals, recognizing safe and competent clinicians, and assessing professionalism and ethics.^77^ However, the exam can be challenging to standardize since follow‐up questions will vary among examiners and by responses provided by candidates. Although examiners are given the same stem questions and criteria for assessing each category, this is still a subjective process in which there is judgment passed by the examiner in evaluating each candidate. Each examiner has differences in discriminatory ability, meaning it can be easier to select the great candidates, but it has been shown that examiners have more difficulty distinguishing poor candidates from average candidates.^78^

A wide criticism of oral examinations is the variation in inter‐rater reliability. Although there are many publications that support this challenge of reliability, Daelmans, Scherpbier, Vleuten, and Donker studied medical students during the end of their clerkship rotation.^79^ The students were given up to ten total exam questions over the course of a week presented from five different examiners. They found that examiners were more reliable when the students being examined had an increased number of questions. They concluded that a single oral exam session was “virtually hopeless in terms of reliability.”^79^

### 4.7 Teaching evaluations

Although the main goal of teaching is for students to learn, it is also helpful to gain feedback about your teaching so that you can continue to grow and improve. The previously mentioned assessment techniques can be helpful to gauge the effectiveness of your teaching through student performance on summative assessments and more frequent responses through formative assessments. If this information is integrated into making adjustments to teaching throughout the class, rotation, or course then you can improve your effectiveness as a teacher.

Teaching evaluations are helpful for providing a different aspect for the educator. The two evaluation techniques described will provide perspectives from two different audiences: other instructors and your students.

#### 4.7.1 Teaching evaluations

Teaching evaluations can be conducted by inviting someone to observe your teaching. Although there may be a formal mechanism for some departments to have a department chair or chief observe your teaching, you can invite others to observe to gain a different perspective. You can ask a peer instructor, a representative from outside your department, or invite a member of the institution's center for teaching and learning if available to observe your class.

It is helpful to have a meeting with the invited observer before the class to discuss your goals, so they know what specifically to observe and schedule a follow‐up session to debrief the observations. Specific topics for the observation can include content knowledge, class organization, methods of engagement, presentation, interactions, and appropriateness of instructional materials.^80^ If it is difficult to have someone attend your class in‐person, you can videotape the class so it can be reviewed. Disadvantages to this technique can often be decreased audio quality, inability to gauge the reaction of students within the class, and inability to capture more active learning techniques if utilized during the teaching.

#### 4.7.2 Student evaluations

The other important perspective is from the learners themselves. It is important to understand their perceptions as students. Course evaluation surveys focus on the input from the students and are typically conducted at the end of the course. These are also known as summative student evaluations of teaching (SETs) and may have an institutional acronym as well.

There is much criticism about the value of student evaluations. However, Benton and Cashin^81^ have shown the validity and reliability of the tool in their literature review and highlight the following misconceptions teachers hold:

1. Students cannot make consistent judgments.
2. Student ratings are just popularity contests.
3. Student ratings are unreliable and invalid.
4. The time of day the course is offered affects ratings.
5. Students will not appreciate good teaching until they are out of college for a few years.
6. Students just want easy courses.
7. Student feedback cannot be used to help improve instruction.
8. Emphasis on student ratings has led to grade inflation

The most important aspect for effective feedback from student evaluations is asking relevant questions to help improve your teaching. Knowing the purpose of the assessment and asking a variety of specific questions in closed and open‐ended question form can be helpful for evaluating your teaching. The University of Wisconsin^82^ provides many helpful examples of questions that can be asked including:

1. The instructor was organized, well prepared, and used class time efficiently.
2. The instructor's teaching methods were effective.
3. The instructor provided helpful feedback.
4. The instructor effectively explained and illustrated course concepts.
5. I could get help if I needed it.
6. The instructor treated students with respect.
7. The text and assigned readings were valuable.
8. Graded assignments helped me understand the course material.
9. I consistently prepared for class.
10. What parts of the course aided your learning the most?
11. What are the strengths of this course?
12. What changes might improve your learning?

In order to optimize the number of participants responding, it is helpful to provide time during class for students to complete the evaluation and explain the rationale behind the evaluation including your value of the feedback.^82^, ^83^, ^84^ Almost all teachers receive negative feedback from students which can feel hurtful when reviewing. It has been suggested to review the comments at a good time for you when you can process the feedback, look for themes in the comments from the students, evaluate if your individual teaching goals were met for the class, and identify key ways that you can improve teaching for the future.^84^

#### 4.7.3 Conclusion

There are many different ways learning can be assessed in the classroom. Evaluate the learning objectives for the teaching to help match the most appropriate assessment techniques to get frequent feedback about how teaching can be improved. In order to assess the effectiveness of teaching, strive to provide a variety of opportunities for assessment to best understand the progress of student learning throughout the class, course, or training experience.

## 5 Residency Mentoring

### 5.1 Overview

In recent decades, growth of the medical physics profession and changes to certification requirements have led to a rapid increase in the number of medical physics residencies. Following demands of the profession and the necessary developments in residency curriculum, quality of medical physics clinical teaching needs to stay on par with the progress of technology and medicine. Given variations in staffing, clinical and teaching workloads and varied institutional support of the clinical residency instructors across the medical physics field, it can be advantageous to arm mentors with proven and effective teaching strategies. The latter is expected to, at least partially, mitigate the effects of variations between clinical institutions and help to produce generations of competent and driven medical physicists.

The American Board of Radiology (ABR)^85^ describes professional functions of a Medical Physicist as follows: “Medical physicists support the diagnosis and treatment of disease through their understanding of the underlying scientific principles of imaging and therapeutic processes.” They use this knowledge to ensure the safe and effective use of radiation for diagnostic or therapeutic purposes. Furthermore, the American Association of Physicists in Medicine (AAPM)^86^ specifies three areas of activity of the medical physicist: clinical service and consultation, research and development, and teaching.

These three aspects of medical physics are critical elements of a clinical medical physics residency program.^87^, ^88^ In addition, recent works point out the importance of interprofessional communication and multidisciplinary approaches to delivering patient care^88^, ^89^, ^90^ across all medical specialties, including radiation oncology, diagnostic radiology, and nuclear medicine.

#### 5.1.1 Who we teach

Medical physics, as a professional discipline, is technologically complex and demands expertise in many scientific and clinical domains. At the same time, it is also a ‘practice’, or profession, and thus demands a set of practical psychomotor skills. Medical physics shares this attribute with such medical specialties as pathology and radiology that, as contrasted with medical physics, have rather minimal patient contact combined with high cognitive demands and technical complexity. Therefore, it is reasonable to explore and adopt some of the educational solutions and recommendations developed for medical residents in these specialties, which have long standing residency training programs. Radiation oncology and radiology residency curricula also have significant areas of overlap with medical physics. These large and long‐standing medical specialty residency programs can and have served as a guide for the more recent transformation of the residency from an alternate to a primary pathway to a career in medical physics.

#### 5.1.2 What we know about how our learners learn

Medical Physics residents, like medical residents, enter residency training with significant prior, mostly didactic, learning experiences. However, most medical physics residents also enter having had some degree of research experience in graduate school, which comes with a different flavor of guidance. As such, residency educators have been actively adopting andragogical (i.e., based on principles of adult learning) approaches^91^, ^92^, ^93^ for their curricular design. Labranche and colleagues^89^ point out that andragogy has been rather successfully used in medical graduate education for some time^94^, ^95^, ^96^ as one approach for facilitating adult learning.^97^ According to Knowles’ concept, adults are motivated to learn through personal life experience so that the learning is primarily self‐directed.^91^, ^92^ Adult learners tend to learn better when the learning process is hands‐on and personally relevant, by being directly and immediately applied to their profession or family life.^89^, ^91^, ^92^

As suggested by Levett‐Jones,^98^ self‐directed learning can be defined as “a process in which individuals take the initiative, with or without the help of others, in diagnosing their learning needs, formulating learning goals, identifying human and material resources for learning, choosing and implementing appropriate learning strategies, and evaluating learning outcomes.”^91^, ^92^ Knox points out that “adults rarely learn, remember or utilize answers for which they did not formulate the question”.^99^

Active learning is rooted in self‐directed learning. When implemented correctly, active learning allows adult learners important autonomy.^100^ Collaborative problem‐solving activities and group discussions result in students being more meaningfully and mindfully involved in the learning process. As a result, knowledge retention increases.^89^, ^98^, ^101^, ^102^, ^103^

To maximize an adult learners’ capacities for absorbing many and a variety of new facts and skills, residents need to be engaged on multiple levels. Some approaches to achieving such engagement are

1. spacing (of teaching, in time, to address limited attention spans and information overload)
2. bringing concrete examples
3. dual coding (presenting text and visuals)
4. interleaving and elaboration (making connections across related subjects while mixing forms of learner engagement)
5. retrieval practice (of the previously learned information)
6. metacognition (self‐reflection on one's learning)
7. generation (independent problem solving)

This overall approach and these techniques were shown to be effective in the rebuilding of a pathology residency curriculum, which led to a twice‐the‐national‐average score on the Residents In‐Service Examination (Rise).^96^ Other approaches that could elicit a multi‐level response are to engage the learner at an emotional or personal level (see Ref. [20] and references therein, e.g., Ref. [21] of this section) and utilize teachers from multiple backgrounds, which has been shown to improve motivation and comprehension.^89^, ^104^, ^105^ Leveraging an emotional connection in learning could be particularly useful in medical physics residency education, and indeed medical education in general, as there is an inherent connection between learners and the goal of the learner: to alleviate suffering through the diagnosis and treatment of disease.

### 5.2 Creating an effective clinical training environment

Although most residency programs have adopted a series of self‐study modules within which to provide clinical training, some programs have also incorporated didactic teachings led either by the faculty or the residents themselves, or both.

Since these learners enter residency training with an advanced degree, they will already possess a substantial knowledge of basic science principles and their practical applications within medical physics. However, both the depth of understanding of didactic principles and the amount of practical hands‐on experience can vary substantially from one incoming resident to another. The goal of the training environment is to ensure that all residents gain proficiency in clinical tasks and that they possess a fundamental understanding of the physics principles underlying those tasks. In other words, the “how to” and the “why it is” are both critically and equally important in the learning environment of medical physics residency.

#### 5.2.1 Self‐study model

Self‐study requires the resident to undertake the responsibility of learning the requisite material for a particular rotation on their own. Virtually all residency curricula include this approach. It works best for candidates who like to learn autonomously and is valuable for fostering independent practice. Residents must be evaluated at the end of each rotation to assure that they have acquired the appropriate breadth and depth of knowledge.^88^, ^106^ The evaluation can be in an oral or written format (e.g., oral exams, discussions, and Q&A sessions). This model reduces the training burden on faculty but may also complicate the process of continuous evaluation and feedback. Such difficulty should be substantially diminished by structuring residency rotations in such a way that includes well defined and frequent mentor‐resident meetings (AAPM Report 249, part 1.7 Resident Evaluation) for periodic knowledge assessment and mandates the resident to maintain periodic reports and logs of their own study and practice.

#### 5.2.2 Didactic lecture model

Lecture requires faculty to be present and engaged with the residents in either a one‐on‐one or classroom style environment. Although “lecture” can mean many things as discussed elsewhere in this document, a key component is that the goals and activity are driven by the lecturer. However, the person leading the lecture could be experienced faculty or even peers. The opportunity for residents to lead lectures not only requires them to approach the material from a different perspective but also develops public speaking skills and prepares them for questions (from experienced faculty) for oral board examinations.

Medical physics residents lecturing of medical students or medical residents can be valuable to both professionals. This sort of near peer‐to‐peer teaching can initiate collaborations and foster increased understanding in broad areas well beyond the topic of the day for both the medical physicist and the medical student or resident.

The format of clinical instruction may change as residents progress through their course of training, gaining more expertise and independence. Residents may spend more time in a didactic and structured mentoring format during the early stages of the program and more time on advanced topics and experiential clinical training during the later stages of the program. The former allows the program faculty increased opportunities for observation, assessment, and guidance, while the latter allows the resident to demonstrate learned skills, initiative, and independence.

#### 5.2.3 Use of nondidactic teaching methods

Clinical teaching is the very essence of medical physics residency education, as the resident transitions from student to independent professional. Problems and challenges of clinical teaching are well‐recognized and described.^107^ In order to combat these challenges, clinical preceptors need to identify and optimize teaching opportunities while performing their clinical work.

Several methods of effective clinical training have gained high recognition among clinical teachers. Many if not all of these methods take an active learning approach and are, in essence, based on a classic concept of “see one, do one, teach one”, or SODOTO. Introduction and development of this concept in US medical education is ascribed to William Halsted and his work on establishing a university‐sponsored, hospital‐based surgical training program at Johns Hopkins Hospital over a 100 years ago.^108^ The SODOTO approach is intuitive, straightforward, and expandable, which explains its longevity and overwhelming popularity for almost a century.^109^, ^110^, ^111^ In recent decades patient safety concerns^112^, ^113^ and a push for standardization,^110^, ^114^ have brought about an evolution of “see one, do one, teach one” to “see many, learn from the outcome, do many with supervision and learn from the outcome, and finally teach many with supervision and learn from the outcome”.^109^

AAPM Medical Physics Practice Guideline (MPPG) 3.b provides guidance on the appropriate levels of oversight when supervising residents in a clinical environment.^115^, ^116^ The three levels of supervision are specified in order of decreasing immediate oversight: Personal supervision, direct supervision, and general supervision. Except in cases in which the resident has demonstrated proficiency through a clearly documented programmatic process, the qualified medical physicist (QMP) should provide final review and oversight, including a signoff, on all clinical work performed by the trainee. A few of the most widely used clinical medical physics training approaches are shadowing, semi‐independent practice, immersion, and simulation.

Basic shadowing consists of observing a mentor's routine clinical work and being gradually allowed to participate. This is by far the most widespread and intuitive teaching method in the clinic. With the “see one, do one, teach one” model, shadowing tends to cover both “see one” and “do one” segments of clinical education, with an emphasis on close (personal) supervision of the trainee. This method has an advantage of added safety when implemented in parallel or following the use of simulated clinical scenarios (see subsection iv).

Semi‐independent practice is a close counterpart to shadowing that allows more opportunity for independence often without direct real‐time guidance but followed closely with mentor review, feedback, and sign‐off. Semi‐independent practice should be a well‐defined component of medical physics residencies, wherein residents are given tasks such as treatment planning,^117^ quality assurance,^115^, ^116^ or chart review^118^, ^119^ only after passing pre‐requisite assessments or otherwise demonstrating a basic level of competency.

Clinical immersion introduces the trainee to the collaborative environment of healthcare, essentially shadowing a team rather than a single mentor. Clinical immersion has been shown to be an effective tool of extended orientation to a new clinical medical physics environment.^120^ Opportunities for immersion abound in both the radiology and radiation oncology clinics. For example, observation of patient plan reviews during weekly chart conferences, or chart rounds, have been recommended for all therapy residencies.^106^ In diagnostic imaging a resident may observe technologists positioning patients and choosing scan parameters. As the number and variety of these observations builds, trainees begin to gain a fuller picture of the operating environment and interactions between colleagues and patients.

Clinical simulation has become an important part of medical and nursing curricula. This approach can not only provide zero‐risk practice of technical skills, but perhaps even more importantly allow for deliberate introduction of failure or error, scenarios. Checking treatment plans with simulated embedded errors has been shown to be valuable for both residents and practicing physicists.^118^, ^121^, ^122^, ^123^, ^124^ Several examples of simulation use can be found in the archives of the Innovations in Medical Physics Education Symposium held annually at the AAPM annual meeting.^125^ Finally, it is important to recognize the increasing complexity and scope of medical physics and design residencies to meet that complexity.^126^ For example, collaboration between institutions and clinical groups with differing capabilities and expertise should be encouraged as it will facilitate access to the widest variety of educational opportunities.

### 5.3 Assessing didactic knowledge, clinical competency, and professional conduct

Under current guidelines from the Commission on Accreditation of Medical Physics Educational Programs (CAMPEP), all incoming residents must either have graduated from a CAMPEP accredited graduate program or completed a CAMPEP approved certificate program, which covers foundational courses required for the field.^127^ This requirement ensures each resident possesses a minimum didactic understanding of concepts prior to beginning clinical training.

AAPM Task Group Report No. 249, “Essentials and Guidelines for Clinical Medical Physics Residency Training Programs”, builds off of the foundation provided by graduate education and provides a framework of core clinical competencies that should be included within the residency training program.^106^ The guidance emphasizes the American Board of Medical Specialties (ABMS) and the Accreditation Council for Graduate Medical Education (ACGME) “core competencies”^128^, ^129^, ^130^, ^131^:

1. Patient care and procedural skills
2. Medical physics knowledge
3. Practice‐based learning and improvement
4. Interpersonal and communication skills
5. Professionalism
6. Systems‐based practice

Both the CAMPEP Residency Standards^127^ and AAPM Report No. 249^106^ provide a comprehensive list of specific tasks and responsibilities to be covered during the course of training. Broadly, however, each of these can be broken into three main components:

1. Didactic and theoretical knowledge
2. Clinical and experimental application
3. Professional and leadership skills

A reasonable goal for training programs is to pair this framework, which applies the theoretical knowledge and competency paradigm to the clinical and professional aspects of the field, with an evaluation system, which benchmarks and quantifies the level of understanding of each resident. Given the breadth and depth of material medical physics residents are exposed to in the course of their training, timely and consistent feedback is paramount to ensuring that key concepts are reinforced and potential gaps in knowledge are rectified.

There are many approaches at the educator's disposal to accomplish these goals. Provided the required material is covered and mastered, the pedagogy each residency program chooses is at the discretion of that program. Common methodologies in the field will be discussed below for each of the first two components above. Development and evaluation of the third component above will be discussed in the following subsection.

#### 5.3.1 Didactic and theoretical knowledge

Board certification is an important indicator of clinical competency, standard of quality care, and ability to practice independently. In North America, certification is provided by the American Board of Radiology (ABR),^132^ American Board of Medical Physics (ABMP),^133^ or the Canadian College of Physicists in Medicine (CCPM).^134^ The final component of the certification process is generally an oral examination. Since this examination will likely represent the final stage of a trainee's demonstration of minimal competence and independent practice, simulated oral board certification examinations are a widely popular mechanism to test the knowledge, training, and clinical competence of the resident. These exams often mimic, to varying degrees, the format and characteristics examinees encounter during oral exams within the board certification process. This assessment approach has been shown to be effective in increasing the confidence of medical residents as they prepare for oral board exams both within radiation oncology^135^ and beyond, including in a virtual environment.^136^ In addition to assessment through examinations within the program structure, external mock oral exams are available to further solidify the resident's knowledge, confidence, and preparation for the certification examination process.

Other approaches to augmenting the didactic and theoretical knowledge of the resident include attending additional graduate classes in related topics or requiring residents to teach and/or present on didactic or clinical topics. Granted appropriate supervision by a QMP,^116^ these methods each provide valuable feedback on the competency of the resident, allowing the supervising staff and faculty the opportunity to quickly and efficiently adjust training while rectifying obvious misunderstandings of concepts. Simultaneously, these further prepare the resident for future responsibilities (written and oral exams, presentations to a peer group, etc.) that they will need to successfully navigate during their career.

#### 5.3.2 Clinical and experimental application

Although a solid foundation in didactic and theoretical medical physics principles and concepts is paramount to becoming a competent medical physicist, the application of that knowledge in effectively identifying and solving clinical problems in a safe and efficient manner requires a complementary, but wholly distinct, set of logical and experimental tools. AAPM TG‐249 states, “The objective of a medical physics residency training program is to educate and train medical physicists to a level of competency sufficient for independent, professional practice in their specified subfield of medical physics”.^106^ Learning these skills in a health care setting, in which an incorrect action can have dire consequences, can be a daunting challenge that requires extensive supervision by experienced individuals. As stated in Subsection 4.2, it is important to define, identify, and document the appropriate levels of supervision for specific tasks performed by the resident.

Of particular emphasis, the QMP is ultimately responsible for the work of the resident. Except in cases in which the resident has demonstrated proficiency through a clearly documented programmatic process, the QMP should provide final review and oversight, including sign‐off, on all work performed by the trainee. Not only is this critical to ensuring a safe clinical practice environment, it is also a necessary consideration in the process of appropriately and effectively assessing the ability of the resident to perform their assigned responsibilities.

Modern medical education training structures have largely moved from time/effort‐based to competency‐based metrics.^137^ Programs that clearly delineate tasks and objectives that must be accomplished and evaluated by qualified personnel are an important process recognized by our profession in evaluating competency. These formalized programs provide a mechanism to confidently ensure that the resident is capable of handling various levels of complexity and independence in accomplishing tasks commensurate with their ability level. Additionally, carefully designed problems or tests can also quickly show deficiencies in the resident's understanding or capabilities and allow for immediate reinforcement of any gaps in knowledge. Such assessment of competence can be difficult due to its relative subjectivity. As a result, the use of entrustable professional activities (EPAs) has become a common approach to demonstrating competency. EPAs are actionable units of work which can be observed and evaluated and provide a practical mechanism to more objectively evaluate competence. The implementation of the EPA concept and careful definition of EPA units in medical physics residency training is the focus of AAPM Working Group on EPA for Medical Physics Residents (WGEPA).

Such evaluations of the resident's capabilities can be performed in both a simulated and real clinical setting, increasing in complexity as the resident completes each required task. Although competency is most commonly evaluated within the context of personal supervision, programs are encouraged to identify useful objective metrics as well. Regardless of the specific evaluation method, the QMP must always be cognizant that the ultimate goal is to ensure the resident is fully capable of independent clinical practice in all required areas of training. This requires a delicate balance of providing the necessary oversight while allowing the resident the increasing freedom they require to reach this goal.

Below are several practical clinical examples of questions and approaches to deploy on a day‐to‐day basis that focus on the aforementioned AAPM Residency Standards and ACGME Core Competencies. To provide further context and connectivity to this Report at large, the also previously mentioned assessment strategies (Diagnostic, Formative, and Summative) provide the backdrop of the shape and context of the questions provided.

- Example 1
  1. Assessment Strategy
    1. Diagnostic assessment example (Before)
  2. CAMPEP residency standard
    1. Didactic and theoretical knowledge (Imaging in radiation therapy example)
  3. ACGME core competencies covered
    1. Patient care and procedural skills
    2. Medical physics knowledge
  4. Question
    1. Which photon interaction dominates in kV imaging? MV imaging?
    2. What is the Z dependence for photoelectric attenuation? Compton attenuation?
    3. How does this factor affect which imaging modality is chosen for setups for RT treatment?
    4. Can you think of a time where MV imaging would be preferred compared to kV for patient setup? If yes, explain how what was discussed above plays into this advantage?
- Example 2
  1. Assessment strategy
    1. Formative assessment example (During)
  2. CAMPEP residency standard
    1. Clinical and experimental application (End to end treatment chain validation example)
  3. ACGME core competencies covered
    1. Patient care and procedural skills
    2. Practice based learning and improvement
  4. Question
    1. How are you marking isocenter on the phantom in the CT Sim and what downstream effects will the precision and accuracy of this mark have on the rest of the end‐to‐end validation?
    2. If you misplace isocenter, what possible problems could occur in the planning and delivery portion of this test?
    3. Describe any advantages or disadvantages of moving the marks to a different location on the phantom.
- Example 3
  1. Assessment strategy
    1. Summative assessment example (After)
  2. CAMPEP residency standard
    1. Professional and leadership skills (pro forma example)
  3. ACGME core competencies covered
    1. Interpersonal and communication skills
    2. Professionalism
    3. Systems based practice
  4. Question
    1. You are employed at a free‐standing, outpatient radiation oncology clinic (Global payments). There is a desire to start a new high‐dose rate brachytherapy program. The program's five‐year prospectus includes the following information:
      1. Volume is projected to be the following for the first year: (Prostate: 3 patients/month, T&O: 8 patients/month)
      2. Patient volume will increase 10% annually for years 2 and 3 and settle to 3% for years 4 and 5
      3. The mix of medicare and commercial payors is 70% / 30%, respectively.
    2. Develop a business plan that demonstrates what the expected revenue is for the HDR program (individual elements and total) for the next 5 years inclusive (i.e., at the end of the 5th year).
    3. What types of department personnel and how many of each are required to provide care for this service?
    4. If each group's salary is $XXXXXXX, at what point in the 5‐year cycle will the treatments become self‐sustaining?
    5. How would you work with the business team in your department to ensure proper staffing and resources (hardware, software, etc.) for the technical team?

### 5.4 Training and assessment in ethical and professional aspects of the practice of medical physics

Training beyond the technical skills required for a clinical career is an important component of a residency training program, specifically including training in ethics, professionalism, and leadership. Training methods and resources for use in incorporating each of these topics into a residency training program are provided here. As these topics have historically not been formally included in the academic or clinical training of current residency faculty members, additional time and effort may be required by the program faculty to ensure that the appropriate level of training is provided.

Ethical practice is an important element of any residency training program. Training in ethical principles should begin by ensuring that program faculty and associated staff create an environment that models ethical behavior. Training by example represents a valuable opportunity to demonstrate these principles in action, and program faculty should take every opportunity to point out practical examples of ethical practice. Recommendations for a comprehensive training program in ethics are provided in “Recommended ethics curriculum for medical physics graduate and residency programs: Report of Task Group 159”.^138^ This document provides resources and references for training in basic ethical principles, examples of ethical encounters, professional conduct, and ethics in clinical practice, research and education. In addition, residents should become familiar with the content of the AAPM code of ethics provided in the report of TG‐109.^139^ Numerous resources are available for training in ethical practice, including purchasable and open courseware from numerous institutions, as well as the Radiological Society of North America (RSNA) and the AAPM Online Ethics and Professionalism Modules.^140^

As with the provision of training in ethical principles, training in professionalism and leadership should also begin with the example provided by program faculty members. Practical examples of professionalism in practice and leadership techniques and strategies should be clearly and deliberately discussed with the resident to illustrate the appropriate learning points. An important resource for providing training in professionalism and leadership is the AAPM Medical Physics Leadership Academy (MPLA).^141^ The MPLA curriculum is divided into three major sections entitled “Personal and Interpersonal”, “Professional and Developmental”, and “Executive and Administrative”, each of which contains a wide variety of useful resources for leadership training and professional development.

Quantifiable evaluation of these skills is difficult as these are by nature subjective domains. However, some physician and medical physics residencies have chosen to regularly solicit and track faculty evaluations of the residents’ conduct. Although such evaluations are also inherently subjective, collecting and analyzing for trends can be a valuable tool for identifying both areas of success and areas in need of improvement.

## 6 eLEARNING

As the healthcare industry becomes increasingly reliant on advanced technologies and data‐driven approaches, the need for highly skilled medical physicists continues to grow. In this dynamic landscape, the adoption of virtual and remote learning methods has emerged as a powerful tool for imparting knowledge and practical skills to trainees and lifelong learners in the field of medical physics. In this section, we delve into the transformative impact of virtual and remote learning technologies, examining their benefits, challenges, and the exciting possibilities they bring to the education of future medical physicists.

eLearning, or e‐learning, is generally considered inclusive of educational systems and terms of instruction comprising technology‐enhanced learning, computer‐aided instruction, distance learning, web‐based learning, internet‐based training, online learning, and virtual learning environments.^142^ Although many medical physics education programs have been leveraging eLearning in some form for many years, prior to the start of the COVID‐19 pandemic, medical physics educators predominantly and preferentially employed tried and true educational approaches to cultivate competent professionals, such as classroom didactic teaching, face‐to‐face in‐person instruction, and in‐person formative and summative assessments. The COVID‐19 pandemic spurred an “emergency transition” and proliferation of eLearning, which has allowed unprecedented number of learners to experience positive and negative effects of eLearning, resulting in several recent reviews of the “new” approach compared to traditional in‐person learning.^143^, ^144^, ^145^, ^146^ According to these studies, the greatest perceived benefits of online teaching platforms include their flexibility, while the commonly perceived barriers to using online teaching platforms include outside distractions and poor internet connectivity.^143^

### 6.1 Learning domains and objectives of eLearning

According to the approach of Bloom and colleagues (“Bloom's taxonomy”^147^), learning can be compartmentalized into three large and interconnected domains: cognitive (factual knowledge, ability to evaluate a case and synthesize a solution); affective (relating to interests, attitudes, and values); and psychomotor (physical skills). Medical physics as a professional discipline is technologically complex in ways that demand cognitive expertise in a diverse spectrum of scientific, industrial, and clinical spheres of knowledge. It is also a decidedly practical clinical specialty that heavily relies on the individual's psychomotor skills and appropriate attitudes. Regarding the affective component of medical physics learning process, first and foremost, human empathy, compassion, and scientific curiosity provide powerful motivation for medical physics learners. However, the affective domain is not limited to motivation. It includes developing correct attitudes for professional practice and ethical behavior in delivering clinical service and contributing to academic research.

Each of Bloom's learning domains can be characterized by a set of learning objectives. Due to inherent and vast differences among the domains of the medical physics learning process, some of the learning objectives are better suited for eLearning instructional medium. Cognitive knowledge can be acquired quite successfully via the eLearning platforms.^143^, ^144^, ^148^, ^149^ Psychomotor skills, on the other hand, are by definition best learned by doing. It follows that the better one replicates real world practice in an eLearning virtual environment, the better prepared our learner will be for their real‐world encounter. In general, medical physics practical teaching encounters follow the four‐stage technique for teaching psychomotor skills:

1. the teacher demonstrates;
2. the teacher demonstrates with commentary and explanation;
3. the learner comments while the teacher demonstrates;
4. the learner talks through the skill while performing it.

One can certainly see how stages 1 and 2 can be implemented virtually.^148^ Moreover, with the help of current videoconferencing methods, it is conceivable that stages 3 and 4 could also be completed remotely. Sheik‐Ali et al.^150^ and Huang et al.^151^ among others have reported on a successful implementation of virtual reality (VR) and augmented reality (AR) tools for skill learning in practice.

An important component of the education for medical physics students and clinical trainees is the development of their professional and ethical behavior in interactions with patients, hospital staff, colleagues, and the public. As commented by Choules,^148^ “attitudes are probably most developed by human interaction, although the principles on which they are based can be learned (and hence, if necessary, taught) by an eLearning ethics or diversity course”. The effect of human interactions, though most impressive if communicating in‐person, can be created via videoconference or phone conversation.

### 6.2 Advantages and disadvantages of elearning compared to face‐to‐face instruction

A thorough understanding of fundamental differences in action mechanisms between eLearning and more traditional ways of delivering educational content will continue to improve learners’ satisfaction with eLearning. eLearning can present several benefits when compared to traditional face‐to‐face learning:

1. eLearning offers flexibility in time and in space.^143^, ^144^, ^145^, ^146^ A learner can be located anywhere from “the 20th row in the lecture theater”.^142^ Synchronous delivery of eLearning materials, in which students participate in classes and learning activities in real time, can foster student engagement, peer‐to‐peer dynamic interactions, and immediate feedback. Asynchronous delivery of eLearning materials, in which students connect with pre‐recorded learning materials on their own schedules, provides students more scheduling flexibility and the ability to set their own pace. Often this format offers increased accessibility.^152^
2. eLearning can be interactive: it can provide 3D visualization, process and event simulation capabilities, and it can be nearly as interactive and efficient as the face‐to‐face learning environment.^142^, ^153^, ^154^, ^155^
3. eLearning can support peer‐to‐peer discussion groups,^154^ near‐peer instruction,^153^ the flipped classroom environment,^156^ improved feedback,^157^ and various videoconference‐based review sessions.^144^, ^155^, ^158^
4. Relative disconnection and anonymity in the eLearning environment may encourage learners to participate freely and openly.^159^
5. From the teacher's perspective, reusability of well‐designed eLearning content may result in a more efficient and targeted application of the preceptor's efforts.^142^, ^148^ The reusability of the content may offer a major advantage for using eLearning in clinical medical physics department settings. The preceptors’ efforts and time can be redistributed to benefit the aspects of clinical education that are most suitable for face‐to‐face, in‐person teaching. This in turn may enhance the quality of a clinical program overall.

Although eLearning offers several advantages to face‐to‐face teaching, it also presents its own challenges. Several difficulties in the widespread adoption of eLearning can be identified:

1. The eLearning environment may result in a less focused and more distracted student audience as compared with in‐person sessions.^146^
2. Students participating in eLearning may find it difficult to balance online attendance with personal and family commitments.^146^
3. Despite the continuing digital connectivity investments, technical difficulties in internet and cellular data access can adversely affect some learners.^142^, ^160^, ^161^ Further, there may be students without access to a quiet, private space to participate in eLearning or without access to the necessary tools, such as a high‐quality webcam or microphone.
4. Learners may struggle with linking theoretical aspects to practice, and eLearning may not afford sufficient real‐life clinical context.^162^, ^163^ Though to a lesser degree than for physician specialties such as surgery or anesthesiology, this point remains valid for medical physics. In clinical medical physics residency, honing one's troubleshooting skills is an essential part of growing into a competent clinical physicist. Close understanding of technical problems, whether related to a device, procedure, or measurement setup, may be, to a degree, compared to the ability of a physician to quickly and correctly diagnose various conditions. Based on multiple studies,^148^, ^162^, ^163^ a blended approach to medical education is more effective than pure eLearning or pure traditional face‐to‐face teaching.
5. Clinical mentors and preceptors may feel reluctant to have trainees bring the information they learned online into a practical clinical teaching realm.^163^, ^164^ Such reluctance is best addressed with thorough understanding of potential reasons and by giving the learner ample opportunity to prove their understanding of a methodology and to demonstrate their critical thinking skills.
6. Educators may have a lack of qualification, expertise, or resources related to eLearning. The expertise in face‐to‐face teaching does not directly translate into a proficiency in eLearning methodologies. Educators often lack resources to develop new skills in teaching in a virtual environment, time, or funding to develop new online learning materials. A survey of medical residency program directors has shown that most programs (79% of 368 respondent programs) do not have a budget for eLearning.^165^ As pointed out by Tabakova,^160^ “from the syllabus to presentation and assessment, components of the eLearning platform need to conform to software features and constraints in terms of space, visual stimulation, and timing”. A simple substitution of in‐person lectures with video streamed lectures is a suboptimal path to creating an effective eLearning platform,^160^, ^164^, ^166^ and this is recognized as a noteworthy challenge for some educators.

As eLearning programs and blended programs incorporating eLearning become increasingly popular, their implementation grows more sophisticated. The number of published reports grows on their successful use, and the significance of the reported negative aspects declines. This is also documented in clinical teaching groups’ reports of successful implementation of VR‐ and AR‐based educational courses.^143^, ^164^, ^165^

### 6.3 Blended learning

With a holistic or integrative view of educational methods, the concept of pure eLearning logically morphs into blended learning, defined as the combination of traditional face‐to‐face learning and asynchronous or synchronous eLearning. In a few recently published meta‐analyses from the medical and healthcare educators’ communities, blended learning was shown to have a consistent, positive impact. Blended learning has been shown to be as effective or more effective than nonblended instruction for knowledge acquisition in health professions.^167^ The conclusions of Liu et al.^167^ were confirmed by Vallee et al.,^168^ based on their review and meta‐analysis of blended learning versus traditional learning in health and medical education. The comparison of blended learning to traditional learning showed significantly better knowledge outcomes for blended learning. Similar results were observed for online computer‐assisted instruction and virtual patient learning support. The meta‐analysis by Vaona et al.^169^ specifically focused on clinical performance results of physicians, nurses, and other licensed healthcare professionals. The study reviewed eLearning versus traditional in‐person learning programs alone and concluded that “e‐learning may make little or no difference in patient outcomes or health professionals’ behaviors, skills, or knowledge.” In a systematic review and meta‐analysis of undergraduate medical education publications from 2000–2017, Pei and Wu^170^ also reported that the learning outcomes in undergraduate medical education, reflected in post‐test scores, pre‐and post‐test score gains, and retention test scores, failed to show statistically significant difference between online and in‐person learners.

In summary, eLearning platforms are increasingly able to simulate real‐world scenarios, providing robust cognitive and psychomotor training remotely. However, it is also recognized that certain aspects of medical physics education, particularly those involving hands‐on practice and the development of professional attitudes, are often most effectively achieved through face‐to‐face interactions. A blended approach, combining the strengths of both eLearning and traditional in‐person methods, has the potential to leverage the benefits of technology while preserving the irreplaceable value of direct, human‐centered instruction.

### 6.4 eLearning and professional development education and assessment

eLearning tools developed for remote classrooms and clinical education have been shown to be effective for continuing professional development. The AAPM held its first all‐virtual Spring Clinical meeting in April 2020, followed by its first all‐virtual annual meeting in July 2020. Pivoting away from in‐person education events, such as large annual professional and scientific conferences, required a massive effort by the organizers from many different fields at the start of the COVID‐19 pandemic—but it also resulted in a positive learning experience for many attendees.^171^, ^172^ With an appropriate virtual learning platform and with buy‐in from all the participants, it is possible to successfully implement a virtual professional collaboration or educational conference. Almarzooq et al.^144^ pointed out that two key roles need to be assigned during synchronous, remote conferences for them to be effective: a moderator to ensure that all the participants can express their opinions, and a knowledgeable technical facilitator to troubleshoot potential software and connectivity issues. Other groups emphasize the importance of flexibility, a strong program of engaging topics, excellent technical support and platforms, high‐quality audio, and the inclusion of re‐recorded presentations as contributing factors to the success of virtual professional conferences.^171^, ^173^

Beyond virtual professional conferences, a major paradigm shift in medical physics has been in remote professional assessment. The specialty medical board organizations that had previously offered in‐person certification exams switched to a virtual environment during the COVID‐19 pandemic. The American Board of Radiology (ABR), which includes medical physics among its specialties, initially postponed exams during the onset of the pandemic. Beginning in 2020, the ABR moved to offer remotely proctored exams to replace the computer‐based written exams (Parts 1 and 2, previously administered through Pearson VUE testing centers) and the in‐person oral exam (Part 3, previously administered at hotels in Louisville, Kentucky and Tucson, Arizona). Although the change and the virtual format presented unique challenges for candidates, proctors, and board staff members, the first remote exams were administered successfully in 2021.^174^

### 6.5 Pragmatic aspects of adding elearning to an education practice

In addition to creating the educational content for teaching online, there are practical aspects of developing eLearning programs that require a significant time commitment, including those related to setting up appropriate logistic pathways for the target audience and managing the eLearning platform. An educator tasked with creating an online course must be ready to invest a significant amount of time in learning the tasks of course management, grading, chat moderation, etc., all using the specific software platform of the institution's choice. Some academic institutions have resources and professional personnel with existing expertise to serve as technical facilitators of the online platform. However, Wittich et al.^175^ noted that 79% of 368 U.S. clinical residency programs do not have a budget for eLearning. This finding suggests that teachers may be required to facilitate online access and troubleshoot technical issues as they arise. Some of these issues might be well out of scope of the actual course content (e.g., incompatibilities of operating systems, failed login attempts, etc.). Solving such issues can be burdensome to both the teacher and the students. Importantly, it could distract from the content of the course. In addition, updating the course content could be a laborious and burdensome task for educators who struggle with technical aspects or do not have access to technical support.

Institutional leaders must work to secure departmental and institutional support for eLearning programs. In practice, as pointed out by Ellaway and Masters,^142^ the relative novelty of eLearning “renders institutions unsure of how to afford the e‐teacher the support and recognition” that they give to their traditional counterparts. Online “performance factors, such as contact hours, academic recognition and advancement” can appear either undervalued or disregarded. One of the most impactful ways for the institution to support eLearning is to hire a technical facilitator for the department or a program to support educational technology. In a concise description by Ellaway and Masters,^142^ educational technologists “act as mediators, facilitators, developers, and enablers for all those working in an educational technological environment… One of the most important roles they perform is resolving tensions between what educators want and what is technically possible and desirable, including the essential option of not employing technology at all.”

In summary, main factors that positively affect eLearning results as pertains to learners’ knowledge, skill, attitude, and satisfaction are the following^161^:

1. interaction and collaboration between learners, educators, and facilitators;
2. careful consideration of learners’ motivation and expectations;
3. the use of user‐friendly, mature technology platforms; and
4. putting learners at the center of the pedagogical paradigm.

Regarding a change in the pedagogical paradigm, as noted by Masters and Ellaway,^176^ learners are relatively autonomous in their use of eLearning and their interactions with the curriculum, mentors, and other program activities (e.g., through discussion groups, interactive simulations, and assessments). This can be easily extrapolated to blended learning, which preserves the majority of the key eLearning features pertaining to knowledge and, partially, to skills and attitudes acquisition. The learner's relative autonomy results in a paradigm shift to active learning, when the student takes the active role of the subject while education itself becomes an object of the educational activity thus morphing into a learner‐centric activity system.^174^ On a very basic level, with the influx of learners’ autonomies, students’ self‐directions, motivations, and self‐organizations determine the success of the educational approach.

A great number of publications describe successful implementation and corresponding learners’ evaluations of the eLearning experience.^143^, ^144^, ^145^, ^146^ Several helpful guideline documents exist pertaining to eLearning in medicine and nursing.^142^, ^164^, ^166^, ^176^ Evidence of the successful adoption of eLearning in medical physics is accumulating. Workshops and other forms of learning opportunities will empower educators in medical physics to create high quality eLearning materials and methods, enabling them to unlock the full potential of the continually evolving eLearning platforms.

## 7 Other Levels of Teaching and Mentoring

### 7.1 Overview

This final section of the WG‐TEACH report describes education practices for a variety of purposes and for reaching a range of audiences and platforms that do not fit clearly into other sections. These topics include working with K‐12 students outside of the classroom, mentoring and advising undergraduate students, science journalism and working with the press, education through social media engagement, and patient education. A collection of AAPM resources is available for physicists interested in embarking on teaching efforts related to these topics, some of which are highlighted in this report.

There are many ways to engage with people outside the profession—opportunities that increase medical physics visibility and that educate the public in what it is that medical physicists do. It is up to physicists to pursue such opportunities, including those that might not seem like obvious choices for furthering the profession. Volunteering for hospital committees, even the radiation safety committee, allows physicists to interact with people who they may not otherwise meet, and get to know other professionals better. Physicists are encouraged to take the time and say “yes” when asked to speak to, write for, or enter discussions with people or groups outside of their usual professional circles. AAPM meetings can be a way to hone presentation skills. From chapter meetings to national meetings, there are often openings for talks from members. Such presentations can function as a steppingstone to conveying messages to others.

Physicists are encouraged, to within boundaries set forth by their employer, to accept interviews from the media, whether through local, regional, or national organizations. The interview experience can be professionally satisfying and may lead to additional teaching and leadership opportunities in the community at large. Enthusiasm for a topic, coupled with engaging communication, encourages real learning—even if the audience doesn't follow all the science.

There are numerous opportunities for physicists to teach in a wide array of environments that do not fit neatly into traditional academic teaching roles, some of which are described here. Many physicists find that seizing these opportunities is both professionally and personally rewarding. Further, engaging in this work may lead to more and more diverse future STEM professionals, and enhance the field of medical physics overall.

### 7.2 Working with K‐12 students outside the classroom

#### 7.2.1 Introduction to K‐12 learning in informal environments

Scientists engage in K‐12 STEM education programs for many reasons, including a desire to support their communities. In addition, K‐12 outreach work may contribute to service and teaching obligations for professional appointments, fulfill outreach or dissemination requirements for grant funding, and improve scientists’ communication skills.^177^ Previous subsections of this report have detailed innovative strategies for student‐centered and active‐learning approaches to classroom instruction. This subsection will introduce strategies for engaging students outside of classrooms, focusing on K‐12 students in informal learning environments.

Although there are several ways to categorize learning opportunities outside of formal class time for this age range, the National Research Council broadly organizes informal science learning into three classifications: everyday learning, designed environments, and educational programs.^178^ Everyday learning experiences encompass a wide array of science learning opportunities that students encounter in settings not explicitly developed for the purpose of STEM teaching. Students gardening, engaging with educational video games, reading online science‐related media, and asking parents questions all represent learning from everyday experiences. Designed environments include STEM learning environments such as museums, science centers, zoos, arboretums, and aquariums. Individual exhibits and demonstrations available in these locations offer more structured learning opportunities. Finally, educational programs encompass a wide range of science learning opportunities including after‐school programs, community science projects, science‐related clubs, STEM festivals, and science cafés.^179^ Although incredibly diverse, all of these different learning opportunities help students build proficiency in STEM, with competencies noted in six common “strands” of learning, as reproduced in Table 2.

**TABLE 2:** Six strands of learning in informal science learning environments (as quoted from the National Research Council^179^).

Learners experience excitement, interest, and motivation to learn about phenomena in the natural and physical world. Learners come to generate, understand, remember, and use concepts, explanations, arguments, models, and facts related to science. Learners manipulate, test, explore, predict, question, observe, and make sense of the natural and physical world. Learners reflect on science as a way of knowing; on processes, concepts, and institutions of science; and on their own process of learning about phenomena. Learners participate in scientific activities and learning practices with others, using scientific language and tools. Learners think about themselves as science learners and develop an identity as someone who knows about, uses, and sometimes contributes to science.

When pursuing these strands of learning, it is helpful to avoid the common pitfall of defaulting to the “deficit model” of education. The deficit model, sometimes known as the “information deficit model,” or “knowledge gap model,” maintains that if students would just fill gaps in knowledge or correct alternative conceptions, then they would make decisions and form opinions consistent with scientific information.^180^ The deficit model posits that these gaps, or deficits, may be addressed when students listen to skilled teachers. That is, if science communication were done better by strong teachers, then students would finally “get it.” This model envisions the scientist educator as the source of knowledge that flows in a single direction toward the learner, reminiscent of “empty vessel” or “blank slate” education theories in which the student acts like a sponge, passively soaking up knowledge directly from the teacher.^181^, ^182^ Research in science communication has shown that students do not become scientifically literate or build scientific competency in these strands simply by exposure to additional scientific information.^183^

The problem with the persistence of the deficit model in the field of science literacy is that most students (including adult students) seldom make decisions based only on scientific evidence or consensus from the scientific community. Instead, they base their scientific understanding on learned scientific knowledge along with a constellation of *other* factors, such as their own goals, previous experiences, identities, values, and beliefs. If educators who undertake K‐12 informal STEM education focus only on filling perceived knowledge gaps, they do a disservice to their students. Instead, research in science education emphasizes the value of leveraging students’ previous science experiences, interest, and ideas; activating their prior knowledge; learning from multiple approaches and with multiple activities; and supporting students as they directly engage in sense‐making, forming evidence‐based explanations, and cooperatively building scientific models.^53^, ^178^, ^184^ These approaches, both inside and outside the classroom, echo guiding principles of a broad K‐12 science education conceptual framework put forward by that National Research Council in 2012.^185^ This framework became the backbone of Next Generation Science Standards first published in 2013 and adopted in some form by several states throughout the US.^186^

#### 7.2.2 A framework for K‐12 science education

The National Research Council and the Next Generation Science Standards describe goals for building proficiency in science by blending three key components or dimensions: scientific and engineering practices, concepts that span across and unite science and engineering fields (“crosscutting concepts”), and core disciplinary ideas in physical sciences, life sciences, earth and space sciences, and engineering. The National Research Council's research‐based framework is based on five key principles. First, science learning is built on foundational principles including the idea that children investigate and evaluate their natural world from a young age. Second, science knowledge is built on core ideas and practices that develop over time when given varied opportunities to learn. Third, science and engineering practice is essential for learning. Fourth, students learn science and engineering best when the subjects are linked to the individual experiences and interests of the student. Finally, all students must be provided with equitable opportunities to learn. Next Generation Science Standards adopted this framework to identify and organize specific learning goals based on grade level and content. Although these standards were originally developed to aid K‐12 schoolteachers, “three‐dimensional learning,” including crosscutting concepts, science and engineering practices, and disciplinary core ideas form an essential framework for anyone involved in K‐12 education, inside or outside of a formal classroom.

#### 7.2.3 Key skills for science communication

The American Association for the Advancement of Science has identified a basic, research‐driven recommended formulation for key skills in quality science communication.^187^ These skills all emphasize aligning educational goals with the intended audience, and are based on the following skill categories:

1. identifying and refining learning objectives;
2. adapting to the expected audience and communication platform;
3. emphasizing, prioritizing, and organizing core messaging consistent with the audience needs;
4. communicating with appropriate and approachable language;
5. using meaningful storytelling for a compelling, logical narrative;
6. designing impactful, accurate, and clear visual displays and data representations;
7. leveraging nonverbal communication to engage the audience and express confidence, openness to co‐learning, and a sense of welcoming;
8. developing a written style that is easy to follow, approachable, and clear; and
9. creating a space for dialogue, including listening, demonstrating humility in the face of historical inequities, and acknowledging privilege.

These skills all can be used to engage audience members and underscore the “so what” of the educational message for the audience. Scientists who are interested in developing these skills for science education and outreach for the K‐12 audience have several training resources available: many universities, museums, and science centers offer science communication classes. Other major scientific societies, including American Association for the Advancement of Science,^188^ and science communication organizations, such as COMPASS,^189^ offer science communication workshops and course series. Some suggestions for practical implementation ideas for this formulation are included in the following section.

#### 7.2.4 Practical suggestions for getting started with informal K‐12 education and outreach

The Portal to the Public Network, supported by the US Institute of Museum and Library Services and the National Science Foundation, has published some recommendations for enabling scientists to engage in activities and educational conversations with K‐12 students.^190^ These recommendations include building enthusiasm about an educational experience, beginning with a “hook,” or simple invitation to participate. Hooks can include asking students to share a story or experience, play a game, complete a challenge, or observe something unusual together with the educator. At the start of an educational event or activity, scientists should introduce themselves, and intentionally seek to discover at what point students are in their understanding of the topic. For example, at an outreach activity discussing day‐to‐day responsibilities of a therapy medical physicist, a physicist might first ask students what they know about radiation and cancer, and then tailor the activity to meet the students at their current level, while making sure the students understand that their ideas are valued. Ideally, conversations should be bidirectional, with the educator facilitating an experience in which students feel educators are engaged in learning with them. Body language and eye contact can help promote a sense of community learning. When designing an informal STEM learning experience for K‐12 students, it is advisable to use straightforward or everyday language whenever possible: avoid jargon, overly complicated words, emotionally charged language, or words with multiple meanings when simpler phrasing works as well. For example, the word “model” has multiple meanings in common use, and not all K‐12 students readily relate to phrases such as “order of magnitude.”

It is often helpful to ask young students questions and wait patiently for answers: incorporating adequate “wait time” is helpful for *all* students, but especially helpful for students in the process of learning English as a second language (emergent multilinguals). An ambitious goal is to facilitate interactions so that the students talk as much as (or more than!) the teacher. When students respond to dialogue with the teacher or each other, students should be prompted in such a way that they are inspired to conduct their own inquiry, define their own questions, and connect new information with their prior knowledge. Instead of simply funneling students to a specific concept or answer, broad questions such as “Have you seen anything like this before?” and “What would you expect to happen next?” can prompt deeper engagement in the learner.^191^ Hands‐on participation also engages students in active learning. Depending on the format, at in‐person events it can be helpful to get a prop into the student's hands as soon as possible during the encounter.

#### 7.2.5 Community science projects

One avenue for K‐12 learning outside the classroom involves community science projects. The National Academies of Sciences, Engineering, and Medicine note these types of projects, widely called “citizen science,” enlist nonprofessional scientists within the community to support the scientific process.^192^ According to the US Federal Crowdsourcing and Citizen Science Act, community science projects represent a “form of open collaboration in which individuals or organizations participate voluntarily in the scientific process in various ways, including enabling the formulation of research questions, creating and refining project design, conducting scientific experiments, collecting and analyzing data, interpreting the results of data, developing technologies and applications, making discoveries, and solving problems.”^193^ Through community science efforts, members of the public partner with scientists by completing tasks such as playing games, tracking COVID‐19 symptoms, counting butterflies, or taking photographs of birds. Beyond crowdsourcing basic data acquisition, community members contributing to these programs may also undergo more specialized training or they may work together with scientists to define problems. Though “citizen science” is the most commonly used name for this partnership, some scientific societies have moved toward using intentionally inclusive language by adopting “community science” as an umbrella term for this type of program.^194^ Other terms for community science include open science, participatory research, crowd‐sourced science, and civic science. Although many large‐scale ongoing community science projects relate to biology, astronomy, and environmental science, successful projects relating to topics relevant in health and medical physics include work in environmental radiation monitoring focusing on the Chernobyl^195^ and Fukushima Daiichi^196^ nuclear disasters or contributing data to train convolutional neural networks to determine MR image quality.^197^

#### 7.2.6 K‐12 informal education opportunities specifically for medical physicists

Dr. Steffel^198^ and van Zyl et al.^199^ recently noted the importance of science communication and outreach for medical physicists. For physicists interested in working with established AAPM groups committed to K‐12 informal education, two AAPM subcommittees specifically developing outreach materials include the Regional Organization Outreach Subcommittee and the Undergraduate Summer Fellowship and Outreach Subcommittee. The Committee on Medical Physicists as Educators and the Public Education Committee both maintain outreach resources. In addition, AAPM supports an official affiliation with the American Institute of Physics’ sister society American Association of Physics Teachers (AAPT), which offers an abundance of resources for physics educators including curricula and lesson plans published in the peer reviewed journal, *The Physics Teacher*. The AAPM's annual meeting offers the popular “Med Phys Wiz Kidz” program for a range of student grade levels,^200^ and AAPM members have previously presented example outreach activities designed for K‐12 audiences in informal learning environments.^201^, ^202^, ^203^, ^204^ These activities provide a resource for physicists to use and adapt in their own communities.

### 7.3 Mentoring and advising undergraduate students

#### 7.3.1 Introduction to advising and mentoring of undergraduate students

Mentors of undergraduate students offer important educational guidance to their mentees, through supervising research, directing internships, hosting job shadow experiences, and advising on a variety of other professional and academic topics. Undergraduate mentoring is a practice that is both complementary to and distinct from formal teaching.^205^ Research indicates that there is a wide variety of undergraduate mentoring programs. Differing characteristics include large variations in mentor training, compensation, their voluntary (or involuntary) nature, frequency and duration of mentoring meetings, and length of mentoring commitment.^206^ Further, mentoring in the undergraduate context has been defined in many different ways in the literature, but an extensive literature review completed by Crisp et al.^207^ noted four common features of these different definitions. First, mentoring relationships center on student development and growth. Second, mentored experiences may take different forms that could include emotional, career, and professional support. Third, mentoring relationships are complementary. Finally, mentors offer their mentees higher levels of expertise in the educational domain. The National Academy of Science, National Academy of Engineering, and Institute of Medicine describe effective mentoring relationships as “characterized by mutual respect, trust, understanding, and empathy. Good mentors are able to share life experiences and wisdom, as well as technical expertise. They are *good listeners*, *good observers*, and *good problem‐solvers*. They make an effort to know, accept, and respect the goals and interests of a student.”^208^

#### 7.3.2 Impact on undergraduate students

Crisp and Cruz^209^ completed a thorough literature review of quantitative and qualitative studies on the effects of mentoring on college student success, and noted the positive impact mentoring experiences have on student persistence, grade point average (GPA), and comfort with the university environment. Students taking part in undergraduate faculty‐mentored STEM research programs self‐report positive impacts the programs have on their lives, including personal and professional gains, laboratory skills acquisition, career and graduate school preparedness, and career and graduate school goal clarification.^210^, ^211^ Additionally, student self‐perceived levels of professional, academic, and general research skills have all been shown to be positively impacted by undergraduate STEM research experiences.^212^ Though top‐performing students who pursue and participate in competitive undergraduate mentored research programs often are interested in graduate school prior to the research experience, participation in these research programs has been shown to *strengthen* this commitment.^213^ Furthermore, experience in these types of programs for students not already at the highest‐achieving level has shown to increase their retention in STEM programs, and also to increase their interest in continuing their education in STEM disciplines.^214^ Undergraduate mentoring programs have been found to be important for retention rates, GPA, and self‐reported gains for members of student groups currently underrepresented in STEM fields, including women, first‐generation college students, and minoritized racial and ethnic groups.^212^

A study across 66 colleges and universities of 2,021 undergraduate students who completed summer STEM undergraduate science internship experiences noted significant self‐perceived gains based on the internship experience, even 9 months post‐experience. These gains included perceived competitiveness relating to future career plans, as well as for characteristics including “independence, intrinsic motivation to learn, and active participation in courses taken after the summer undergraduate research experience”^215^ Science students in a 2016 study of UK undergraduate students similarly reported increased self‐perceived gains in key professional skill categories including time management, working independently, and ability to interpret results and read primary literature following completion of a STEM‐based undergraduate internship experience.^216^ Long‐term gains for students, years after the conclusion of undergraduate research experiences, include confidence‐building in the ability to conduct research and learning of laboratory techniques.^217^

#### 7.3.3 Characteristics of quality mentors for undergraduate students

Mentoring undergraduate students offers benefits to the mentor as well as the mentee. In addition to the intrinsic satisfaction that many mentors find from their work, mentoring undergraduate students encourages STEM professionals to develop and recruit top students, sharpen teaching skills, and stay abreast of current developments in their fields.^208^ Aspects of quality mentors vary with the program, student, and types of relationships^218^; however, the literature review from Crisp et al.^207^ identified common characteristics of effective mentors across multiple studies. These characteristics include mentors who encourage, motivate, and advocate for their mentees; who support students to reach reasonably high expectations; and who relate to students on an emotional level, with a personal commitment to mentee success. Many undergraduate STEM research programs pair an undergraduate student with a single experienced faculty mentor in a one‐on‐one setting. Kobulnicky and Dale^219^ advocate instead for a community mentoring model in which a community or network of mentors coalesce to meet the variable and numerous needs of individual mentees.

#### 7.3.4 Mentoring a job shadow or observership experience

Pre‐medical, pre‐pharmacy, and pre‐nursing undergraduate students routinely complete job shadow or observership experiences as part of their preparation for professional school and its application process. Studies examining formal pre‐medical job shadow programs have shown that undergraduate alumni of these programs feel more familiar with physicians’ day‐to‐day responsibilities and also more familiar with patient‐physician interactions,^220^, ^221^ though traditional shadowing experiences can vary significantly.^222^ The Association of American Medical Colleges’ 2013 guidelines on shadowing experiences^223^ offers a valuable resource for basic requirements of a successful shadowing experience. These guidelines stress that students must first be educated on the Health Insurance Portability and Accountability Act (HIPAA), with an emphasis on patient rights, confidentiality, and privacy. Students must understand the basic code of conduct for the organization as well as salient expectations and requirements, such as immunization history. Further, shadowing students must never actually engage in practicing medicine, and they must respect boundaries regarding patient consent. Other recommendations for successful shadowing experiences, for both the student and the host, are described by Irby et al.,^224^ including the necessity of adequate preparation, clear learning objectives, and sufficient follow up. All these foundational requirements and recommendations have clear parallels for undergraduate students interested in shadowing clinical medical physicists at work.

#### 7.3.5 Supervising a research or internship experience

Pita et al.^225^ have suggestions on best practices for acting as a faculty mentor to supervise undergraduate research and internship experiences. These recommendations include the importance of carving out time for mentorship, building a sense of community, fostering attentive and clear communication (including about mutual expectations), understanding student inexperience and competing commitments, and encouraging students to present their work. Shellito et al.^226^ echo many of these sentiments, adding the importance of developing well‐defined projects at the students’ level(s); getting to know students personally; respecting mentees as colleagues; building opportunities over time to assist students in growing toward independence; and being welcoming, friendly, and supportive. The literature review performed by Shanahan et al.^227^ summarized what they considered ten key practices of quality undergraduate research mentors consistent in the literature, reproduced in Table 3 below.

**TABLE 3:** Ten salient practices in undergraduate research mentors (from Shanahan et al. 2015).

Perform strategic pre‐planning in order to be ready to respond to students’ varying needs and abilities throughout the research process. Set clear and well‐scaffolded expectations for undergraduate researchers. Teach the technical skills, methods, and techniques of conducting research in the discipline. Balance rigorous expectations with emotional support and appropriate personal interest in students. Build community among groups of undergraduate researchers and mentors, including graduate students, postdoctoral fellows, and any other members of the research team. Dedicate time as well to one‐on‐one, hands‐on mentoring. Increase student ownership of the research over time. Support students’ professional developments through networking and explaining norms of the discipline. Create intentional, laddered opportunities for peers and “near peers” to learn mentoring skills and to bring larger numbers of undergraduates into scholarly opportunities. Encourage students to share their findings and provide guidance on how to do so effectively in oral and poster presentations and in writing.

Literature suggests that undergraduate participation in formal scientific writing and presentation, via publication in peer‐reviewed journals, grant writing, or through meetings and conferences, has a significant positive impact on undergraduate students and highlights the importance of the student‐mentor co‐research.^228^, ^229^ Physicists interested in mentoring students in internship and research experiences are encouraged to review the AAPM's Code of Ethics,^230^ including recommendations on education and publication ethics.

#### 7.3.6 AAPM's undergraduate summer programs

The most specific published work regarding undergraduate STEM internships and research experiences relates to AAPM's sponsored summer undergraduate research programs. These programs include the Summer Undergraduate Fellowship Program (SUFP) and the Diversity Recruitment through Education and Mentoring Program (DREAM), formerly the Minority Undergraduate Summer Experience Program. AAPM began offering undergraduate summer fellowships in 2001 and has continued to offer opportunities for undergraduate engagement in medical physics every year since, including two years of virtual fellowships in 2020 and 2021 during the COVID‐19 pandemic. Both the SUFP and DREAM programs are highly competitive, and pair exceptional undergraduate students with mentors working in medical physics for a 10‐week paid summer internship experience focused on medical physics research and/or clinical work. Surveyed mentors and student fellows expressed satisfaction with the AAPM programs (the majority noting they were “very satisfied”), and 65% of surveyed past fellows reported that they decided to pursue graduate school in medical physics based on their experience in the AAPM programs. At the time of the survey, 70% of program alumni were currently involved in the medical physics field as graduate students, residents, or professionals, and the majority of these alumni had published or were in the process of publishing original medical physics work.^231^ Reported possible avenues for improvement of the programs included increasing funding to allow for additional students to take part in the programs, increasing the geographical locations of potential host institutions, and developing a robust tracking system of student alumni and previous research topics. Physicists interested in hosting SUFP or DREAM students may apply through the AAPM's grants and fellowships web page.

### 7.4 Science journalism and working with the press

Communicating scientific information through news media, either by directly crafting scientific media for the adult public outside of the field of medical physics, or by working with scientific journalists through interviews, can be an effective way for physicists to publicize research findings, promote clinical developments, and otherwise share their work with a wider audience. Major science news outlets such as *Scientific American* and *Popular Science*, the science section of publications such as the *New York Times*, and the news sections of scientific journals such as *Nature* and *Science*, all regularly publish timely and engaging scientific content, and a myriad of other media options exist for communicating science to many different types of audiences. The Pew Research Center recently found that general news outlets are the largest providers of science news to Americans, and that about one third of Americans consume science news multiple times per week,^232^ including through websites, podcasts, television, radio, newspapers, or a combination of sources. Strategic partnerships with news media therefore have the potential to advance the field of medical physics while disseminating information. AAPM's Code of Ethics^233^ notes that AAPM members who are “communicating to the media or public via any means should clearly state whether the information provided is based upon scientific studies, expert consensus, professional experience, or personal opinion.” Further basic ethical considerations involve accurately representing data without excessive “hype,”^234^ respecting research embargo schedules,^235^ and avoiding supporting predatory journals.^236^

Beyond basic ethical considerations, elements of quality science writing and communication involve explaining sometimes complicated findings simply, accurately, and with compelling storytelling.^237^ It is also important to understand the specific audience and communicate at an appropriate level given the readership. The National Association of Science Writers (NASW), the American Association for the Advancement of Science (AAAS), and The Open Notebook all offer guidance on getting started with science journalism and working with the press. For interested readers, some examples of quality science writing related to medical physics include science writer Dr. Catherine Steffel's reporting in *Physics World* on “Proton radiography: one step closer to clinical use”^238^ or Dr. Dave Jordan's “State of the art in magnetic resonance imaging” contribution to *Physics Today*.^239^ Physicists who are interested in sitting for interviews or otherwise liaising with media professionals may note Dr. Rebecca Milman‐Marsh's work on publicizing the joint professional society recommendation to discontinue the routine use of gonadal and fetal lead shielding during imaging procedures, such as her contributions to a news story reported by Good Morning America^240^ and the *New York Times*.^241^

Physicists interested in crafting original writing themselves should begin by defining the story, audience, and platform of their desired publication. These decisions will shape the scope, detail, focus, format, and style of the piece. Authors should properly research and vet sources.^242^ They should use approachable and active language with a clear story (as opposed to a list of facts).^243^ Physicists familiar with research, presenting, and publishing are well aware of the gravity of reporting robust science. The same holds true for science writing in all forms: science writers must evaluate statistics, sample size, confidence levels, uncertainty, avoid confusing causation with correlation, etc., in reporting on their own work as well as in reporting on the work of others. Special cautions to take when undertaking health reporting include such practices as avoiding the use of the word “cure,” and noting potential gaps or limitations in research, especially with respect to race, gender, and/or potential conflicts of interest.^244^

Physicists who are hoping to work with press professionals may have access to their organization's press office, which can be a valuable resource in crafting an effective press release, organizing a news conference, or developing a media strategy. Frequent communication with members of the press office can be valuable. Following a press release, scientists should expect to be available to respond promptly to reporter inquiries, and note that journalists often work with inflexible, demanding deadlines that generally require faster response times than other communication professionals.^245^ When working with journalists, maintain a focused and concentrated primary message, a professional but friendly attitude, and as with quality science writing, always try to convey robust science using simple, approachable language free of jargon, keeping the work short and to the point.^246^ Individuals working with journalists should always assume everything is on the record,^247^ (at least until mutual trust is established with a given journalist) and be aware that conversational interactions, especially ones that leverage quality graphics or video depictions, are often effective ways to communicate.

### 7.5 Education through social media engagement

#### 7.5.1 Social media introduction

The introduction, and subsequent rapid societal implementation, of social media has fundamentally changed the way scientists and scientific educators communicate, promote work, recruit, and share ideas. Social media has moved scientific communication and education from something done in a dark windowless laboratory and dingy auditorium to a mainstream occurrence that the average person can interact with, discuss, and learn from experts. Social media is a space that provides an unparalleled ability to voice opinions, share information, and create scientific discussions at a rapid pace—but can also foster misinformation, hostility, and public confusion if not done ethically and properly.^248^ As a tool, social media can improve the reach and impact of research,^249^, ^250^ improve the ability to recruit prospective students and faculty,^251^, ^252^ influence public policy,^253^ and improve education.^254^, ^255^ To create an effective social media strategy, physicists must address some key questions before getting started: What is the best platform to disseminate this information, who is the intended audience and what is the vision or desired outcome of posting? Table 4 offers a brief introduction to these concepts, with specific attention to implementation in medical physics and medical physics education.

**TABLE 4:** Summary of selected social media options.

Social media platform	Content type	Platform specifics	Notes for physicists
X (formerly Twitter)	Multimedia platform (text, images, videos, etc.)	280‐character messages (a.k.a. tweets or posts) Handle/Username—how a user is identified on the platform. A handle is always preceded immediately by the @ symbol. (i.e. @AAPMHQ is the main account for AAPM). Bio—A user's bio is a short (up to 160 characters) personal description that appears in the user's profile that serves to characterize your persona on X/Twitter. #hashtags—A hashtag is any word or phrase immediately preceded by the # symbol. When a user clicks or taps on a hashtag, they will see other Tweets containing the same keyword or topic. Mention—Users mention other accounts in tweets by including the @ sign followed directly by another username is called a “mention”. “Mentions” also refer to tweets in which your @username was included. · Retweet—The act of sharing another account's Tweet to all of your followers.^256^	Users create a handle and follow any accounts that interest them. Follow @AAPMHQ for the latest information from AAPM. Medical physicists can engage with other MPs on topics of common interest.
Facebook	Multimedia platform (text, links, photos, video, live streaming, profiles, groups, and events)	Profile—A user's personal description that is visible publicly or to specified users. Include name, picture and any desired personal information. Friends—people or groups that a user chooses to follow and allows to see their profile and information. · Pages and groups—Defined groups, managed by a user, for people with similar interests. Anyone can follow a page of Facebook; groups on Facebook can require that members meet certain criteria to join.	Great for schools, residencies and labs to keep present and previous members up to date on current information. Pages/groups allow users to engage without having to use a personal account.
Instagram	Images and video	Image‐based platform, with extensive photo editing functionality, that allows users to post 1–10 photos/videos at a time with text captions. Images and videos are stored within a feed/profile and are shown in chronological order. Story—An image or series of images that only appear on a user's profile for 24 h. · Live streaming—Live video visible to followers.^257^	Good option for groups or labs with visually diverse work. Great way to promote a campus or department. Ability to cross post to Facebook allows for posting on multiple outlets at once.
Youtube/Vimeo	Video‐ and streaming‐based platform	Video‐based platforms that allow individuals and organizations to upload, view, rate, share, report, and comment on videos. Subscribe—Users can subscribe to other users and channels and be notified when that user or channel uploads new content. ·Universities and other research institutions often use YouTube for educational and/or PR purposes. Although there is a pay version of YouTube, the free version contains advertisements.^258^	Great for educational and PR videos that may interest a wide range of people. Great way to create a repository of educational modules or materials that students can access at any time.
Reddit	Multimedia platform (text, links, photos, video, live streaming, profiles, groups, and events)	Discussion‐based platform that allows users (designed with “u/” and then a username) to engage in groups and discussion forums. Subreddit‐r/—A defined group in the reddit community with moderators that help regulate the information to be specific to the group. · AMA – Ask Me Anything—An informal question‐and‐answer session that has become a popular way to engage with users on specific topics.	Follow and engage with r/MedicalPhysics. Hold an AMA for your school or lab, and allow prospective students to ask questions about life in your program or lab
LinkedIn	Professional networking platform (text, links, photos, video, live streaming, profiles, and company pages)	Connections – Direct, professional connections on LinkedIn work similarly to adding friends on other social media platforms, creating a network of peers and colleagues. Company and institution pages – Organizations can create dedicated pages to share updates, job listings, and other institution‐specific news. · Groups – Discussion forums for professionals to network, discuss industry topics, and share insights.	Join professional groups such as the American Association of Physicists in Medicine to stay updated on discussions in the field. Use LinkedIn Posts to write short, accessible summaries of recent publications or educational insights, and link to full articles and other resources to increase visibility.

#### 7.5.2 Vision, audience, content, and delivery

Social media offers a unique avenue that allows users to engage with a variety of different target audiences but can also be intimidating to start and even harder to understand how to reach people. Want to promote a new learning technique? Post the resources and content and @ (“at” or tag) educational and medical physics experts to increase engagement. Want to start a discussion on a potential public policy change? Create a group on Facebook promoting a change you would like to see happen and invite interested members to join. Interested in learning more about a specific learning approach or implementing a new teaching strategy? Through personal or direct messages, users can engage in 1‐to‐1 discussion with an interested expert and get helpful resources or information. To do this can be very daunting to start and difficult to maintain—but as with most things, practice and perseverance pay off. When beginning with social media, it is important to determine the key factors that will influence social media strategy: vision, audience, content, and delivery.

An important aspect of beginning with social media and developing a social media strategy, especially in medical physics, is to determine who is the intended audience, how do users want to interact with the audience, and what is the vision, or desired outcome, for the interaction. Most social media platforms were designed to help facilitate forming connections and reach people with a specific and (hopefully) thought‐out message. Defining the audience will help define content, which will make the content more specific and applicable. Specifically curated content tends to resonate better with the audience and allow them to better receive the specific intended information or vision. Delivering curated content will allow you to reach more people while also controlling how this content is present. Vision, audience, content, and delivery are key to social media success and engagement.

It is important to remember that, with social media, users can't control how content is received but can control how information is presented. This allows the unique opportunity not only to promote individual scientists, but also promote research, new ideas, interesting educational methods, requests for collaboration, and even influence public policy. Social media provides the ability for users to engage directly with any desired group (but knowing who is targeted is just as important). Professional archers can have the best equipment available to them, but if they can't see the target or know where it is they may never hit it. The same concept applies with social media; users can have amazing content but fail to reach the intended audience. Additionally, using the wrong arrow or the incorrect bow can prevent the archer from achieving the goal of hitting the bullseye. With social media, content becomes the arrow and platform becomes the bow. Understanding the best avenue for a specific type of post is challenging, even for seasoned social media influencers, but is something that can be learned and practiced. By understanding the vision, audience, content, and delivery, social media can become a key part of educational promotion, recruiting, and collegial engagement.

The following is an example of this strategy in action for a new medical physics laboratory looking for a postdoc. Dr. Photon, a medical physicist, embarks on establishing a social media strategy to attract high‐quality postdoc candidates for their newly launched medical physics laboratory. They begin by clarifying their vision: to promote their lab and position, driving interest and applications. Identifying the target audience, they aim to engage postdocs with a medical physics background and an interest in clinical implementation, along with the broader medical physics community. To achieve their goals, Dr. Photon decides on three key platforms: Facebook, X (formerly Twitter), and Reddit.

On Facebook, they create a dedicated group for their lab, sharing general information, updates, and formal job postings. They invite current lab members and colleagues to follow the page. On X, they set up an account for their lab, strategically following related accounts and posting an image of their lab space along with a link to the job posting. To foster interactive discussions, Dr. Photon conducts a Reddit AMA, inviting questions from interested individuals. They continue engaging with applicants and utilize the social media accounts to promote their lab's work even after successfully hiring a postdoc. By aligning their vision, audience, content, and platform selection, Dr. Photon effectively leverages social media to achieve their recruitment goals and promote their research endeavors.

When implemented correctly, social media can improve the reach and impact of research, improve the ability to recruit prospective members, influence public policy, improve education, and allow the user to engage on an international scientific stage. Creating an effective social media strategy can be complicated and takes practice, but can be simplified by remembering the key questions of first, what is the vision, second, who is the audience, third, what is the best platform to disseminate this information, and finally, what is the appropriate content?

### 7.6 Patient education

Oftentimes, most medical physicists have limited contact with patients and their families except when working on special procedures that require face‐to‐face interactions with the patient. However, the medical physicist's role in the education of patients and their families in both diagnostic and therapeutic areas has been growing and changing. Some, particularly in the therapeutic physics community, envision a more proactive role in patient education by the physicists,^259^ a role that would increase physics visibility while decreasing the misunderstanding of our role in both diagnosis and treatment of patients. Patients often come to radiology, nuclear medicine, and radiation oncology with a fear of radiation and many misunderstandings about what it is that radiation can and cannot do.^260^, ^261^, ^262^, ^263^ Atwood et al.^264^ led a randomized prospective phase III clinical trial examining the impact of physicist‐patient consultations, and found that radiation oncology patients who took part in the consultation program had significantly decreased anxiety and increased satisfaction scores compared to those in the control group. Medical physicists, with specialized knowledge, can help to bridge the understanding gap and help patients to be less fearful and better informed throughout the diagnosis and treatment process.

With this increased role in patient and family education comes the need to understand how to speak to patients, their families, and/or caregivers. As it is not a role that has been traditionally at the forefront of medical physics specialties, there is some amount of education that is necessary to become proficient in communication with patients and their families. Physicists can learn from physician, nursing, and technologist colleagues who have a wealth of experience in this arena.^264^, ^265^

King and Hoppe in their review “‘Best Practice’ for Patient‐Centered Communication: A Narrative Review” states that best practice for communication in medical encounters fosters a relationship, gathers information, provides information, makes decisions, responds to emotions, and enables disease‐ and treatment‐related behaviors.^266^ Let us break down how professionals in physics can help reach these goals.

First, foster a relationship. Physicists should introduce themselves to the patient/family/caregiver with an explanation of what they do and why they are there. They should make eye contact while speaking and have an awareness of body language and the effect it can have on rapport with the patient, their family and/or caregiver(s). Berman and Chutka in their paper “Assessing effective physician‐patient communication skills: ‘Are you listening to me, doc?’” express the importance of nonverbal communication skills, as body language and paralinguistic clues make up about 93% of communication.^267^ Body language is an indicator to the patient of the communicator's feelings. Nonverbal communication can convey warmth, caring, empathy, reassurance, and support on the part of the professional they are communicating with, or it can convey the complete opposite: disinterest, boredom, anger, irritation or disbelief. The former can make the communication productive, leading to patient satisfaction and compliance, and the latter can make communication unproductive, leading to patient dissatisfaction and noncompliance. Maintain eye contact throughout the conversation. Lean toward the patient from a comfortable distance. Demonstrate concern for the patient.

Next, gather information. Ask questions to assess the level of understanding of the patient, family and/or caregiver. Questions asked should be, where possible, both open‐ended and understandable. When asking these questions, use patient‐centered techniques and allow sufficient time for the patient to express their thoughts. Permit the patient to speak without interruption, listening actively at all times. Make sure the patient has sufficient time to speak completely. Take the time to listen carefully to their questions and concerns before speaking. Physicists should keep in mind that some patient questions may not be related to the physicist's area of expertise, and it is totally acceptable to inform the patient that someone else may be a better person to ask about a specific topic. Try to keep the patient productively focused on the questions that are relevant to the topic at hand and stay focused.

In providing information, make sure to give explanations using plain language. Many medical fields, including medical physics, tend to use a lot of acronyms and jargon. Try to avoid using either, as both may cause confusion and misinterpretation.^260^, ^263^ If acronyms and jargon are absolutely unavoidable, then take the time to explain the terms fully the first time they are used to limit misunderstandings, and be sure that when they are used again that the patient understands. Use analogies to everyday life whenever possible to help patients and their families understand key concepts. For example, instead of using the term “erythema,” explain that radiation may cause the skin to redden like it would with a sunburn for patients who have experienced sunburns, or like a friction or rug burn for patients who have not experienced sunburns.

Effective communication makes decisions and decision‐making for the patient and their families easier. Explanations to patients’ questions should help patients in making decisions about their care and clear up doubts. Where possible, attempt to respond to emotions by acknowledging and allaying fears. Express sympathy, empathy, and reassurance. Facilitate a patient's emotional turmoil when possible, and let others know of any psychological distress that should be addressed.

Information communicated during patient consults should be helpful to the patient, enabling positive disease‐ and treatment‐related behaviors. In order for that to happen, the patient must understand what is being related to them. Ensure that patients understand the ideas and concepts discussed by asking them to explain back what has been conveyed. This should help in assessing the patient's level of understanding and determine if the patient's questions have been answered adequately.

Practice speaking to patients; for most people, it is not a natural skill. There are programs that help medical physicists learn these skills,^268^, ^269^ though there is more guidance for physicians and paraprofessionals.^265^, ^266^, ^267^, ^270^ Setting the foundation of these skills in graduate programs and emphasizing them in residency programs becoming more important as direct patient interaction becomes more prevalent.

### 7.7 Nonphysics health professionals

One type of teaching that has, to this point, been neglected is that of educating nonphysics health care professionals. This would include both allied health professionals such as nursing as well as medical professionals in specialties outside of those that traditionally both require and receive medical physics training. The European Federation of Organisations for Medical Physics (EFOMP) has published extensively on the topic of education for nonphysics healthcare providers^271^, ^272^, ^273^, ^274^ up to and including the EFOMP policy statement 18 on medical physics education for nonphysics healthcare professions.^275^ Allied health professionals’ and referring providers’ level of background and education in medical physics ideas can drastically influence both patient care and patients themselves.

Ohno and Kaori note that nurses can ease patients’ minds, but also can increase their unease about radiation depending on their biases and education level about the subject matter.^276^ This is also true of other healthcare professionals, especially referring providers. With that, there is a push, at least in Europe, toward better radiation and radiation protection training of doctors in all specialties (not just those that use radiation)^277^ as well as medical students.^277^, ^278^ This can be an opportunity for the medical physicist to serve as an expert in this area.

The EFOMP policy statement 18^275^ emphasizes the importance of understanding the specific needs as to medical physics content for the group of providers being educated. Understanding the background of the healthcare professionals that will be taught with respect to their physics knowledge is also valuable. Finally, the educator needs to know the role the professional plays in relation to medical physics. These three tips can be summarized by knowing your audience – something that has been stated before in this document. Knowing the audience plays an important role in determining which pedagogical tools previously presented could be most effective in successfully communicating medical physics related topics to these groups.

So how can medical physicists teach such a wide range of professionals? EFOMP has designed a curriculum that can be used in teaching nonphysics healthcare professionals and can be adjusted for different levels of background and understanding of the topic.^271^ They also stress the importance of keeping the level of physics‐knowledge detail to the level in accordance with the legal clinical role of the healthcare professional being taught.^275^ Active learning strategies presented previously can be used in the education process of these individuals. Once the audience has been properly identified, strategies can be built from the various techniques, depending on the audience and educator preference. Better education of nonphysics healthcare providers can help to raise awareness among the professions as to the work that medical physicists do. Such education can also help our patients to receive the most appropriate care.

## 8 Summary and Conclusions

In this report we distinguish between *teaching* and *mentoring* while acknowledging the strong overlap between the two. By “teaching” we tend to mean explaining principles, concepts, or formalism within a classroom setting or by means of written expression, while by “mentoring” we primarily mean more of a hands‐on coaching role within the clinical setting of a residency program. This report has presented an overview of various types of approaches to the teaching and mentoring of medical physics, from the more traditional classroom teaching and clinical mentoring to other aspects of teaching, such as e‐learning and communicating with the public. It is important to keep in mind that this report does not presume to present how everyone involved in the teaching and mentoring of medical physics should approach what they do in the classroom, the clinic, or in their communications with those outside the medical professions. Rather, the approaches presented here are examples of teaching methods that have been demonstrated to work for many educators in numerous situations. It is up to the individual educator to be aware of best practices and to attempt—even on a small scale—changes that might improve their teaching.

It is interesting to note that, as scientists, we pride ourselves on basing our approach to our work on the published work of others who came before us. We develop the way we think about work‐related topics by continually educating ourselves on the results of research—that is, our approach to what we do is done *scientifically*. There is one aspect of our work, however, where that is often not the case: that aspect associated with teaching and mentoring.

It is well known that teachers tend to teach the way they were taught, and they mentor the way they were mentored—most often by lecturing to the students in the case of teaching and providing written instruction or simply demonstrating how something is done in the case of mentoring. This is not because it has been shown in Education Research that lecturing and demonstrating are the best ways to approach teaching and mentoring—quite the opposite—but rather because that is the way it has been done in the past.

It is well known from Physics Education Research that, compared to passive lecture, active engagement of the students in classroom teaching can improve student understanding of concepts and their ability to solve problems.^6^ This does not mean that lecture needs to be abandoned, but rather that it needs to be reconsidered and adjusted if we wish to continue lecturing while taking a *scientific approach* to our teaching. It is also well known that taking an active‐learning approach to mentoring adult learners such as medical or medical physics residents can help improve retention.^89^, ^103^

This report has presented and discussed various approaches to different kinds of teaching leading to active learning, including applications to didactic classroom teaching and clinical and research mentoring. It is encouraging to see that, as a result of AAPM teaching workshops, numerous medical physicists have already adopted many of the active‐learning techniques discussed in this report, as evidenced by the annual abstract submissions to the Innovation in Medical Physics Education session.^279^ Nevertheless, more work needs to be done in promoting research‐driven teaching techniques in the medical physics classroom.

An issue often brought up in the discussion of incorporating alternative teaching techniques into the teaching of medical physics is *time*—it does take time and effort to change the way one approaches one's teaching and mentoring. Although it is true that teaching itself does not directly pay for the work done in the clinic, the conclusion that it is not worth putting time into teaching does not make sense. Improving the effectiveness of teaching is an investment in the future of the field of medical physics; it can improve the quality of students graduating from a medical physics program, increase the passing rate on the ABR exams, improve the reputation of a graduate program, and improve the quality of prospective students attracted to that program. This means that it can help improve patients’ lives. It is an investment in our patients as well as in our students, the next generation of medical physicists. It is the right thing to do.

## AUTHOR CONTRIBUTIONS

All of the authors listed for this report have made substantial and direct contributions to the writing of the report.

## CONFLICT OF INTEREST STATEMENT

The Chair of the Working Group on Teaching Educators and Clinicians How (WGTEACH) has reviewed the required Conflict of Interest statement on file for each member of the Working Group on Teaching Educators and Clinicians How and determined that disclosure of potential Conflicts of Interest is an adequate management plan.

All members of the Working Group on Teaching Educators and Clinicians How attest that they have no potential Conflicts of Interest related to the subject matter or materials presented in this document.
